# Noncoding variation near *UBE2E2* orchestrates cardiometabolic pathophenotypes through polygenic effectors

**DOI:** 10.1172/jci.insight.184140

**Published:** 2024-12-10

**Authors:** Yang Zhang, Natalie L. David, Tristan Pesaresi, Rosemary E. Andrews, G.V. Naveen Kumar, Hongyin Chen, Wanning Qiao, Jinzhao Yang, Kareena Patel, Tania Amorim, Ankit X. Sharma, Silvia Liu, Matthew L. Steinhauser

**Affiliations:** 1 School of Public Health (Shenzhen), Sun Yat-sen University, Shenzhen, Guangdong, China.; 2 Aging Institute, University of Pittsburgh School of Medicine, Pittsburgh, Pennsylvania, USA.; 3 Division of Genetics, Brigham and Women’s Hospital, Harvard Medical School, Boston, Massachusetts, USA.; 4 Department of Human Genetics, University of Pittsburgh, School of Public Health, Pittsburgh, Pennsylvania, USA.; 5 Division of Endocrinology and Metabolism, Department of Medicine, University of Pittsburgh School of Medicine, Pittsburgh, Pennsylvania, USA.; 6 Department of Pathology, University of Pittsburgh School of Medicine, Pittsburgh, Pennsylvania, USA.; 7 Pittsburgh Liver Research Center, University of Pittsburgh, Pittsburgh, Pennsylvania, USA.; 8 Division of Cardiology, Department of Medicine, University of Pittsburgh School of Medicine, Pittsburgh, Pennsylvania, USA.

**Keywords:** Endocrinology, Genetics, Adipose tissue, Diabetes, Genetic variation

## Abstract

Mechanisms underpinning signals from genome-wide association studies remain poorly understood, particularly for noncoding variation and for complex diseases such as type 2 diabetes mellitus (T2D) where pathogenic mechanisms in multiple different tissues may be disease driving. One approach is to study relevant endophenotypes, a strategy we applied to the *UBE2E2* locus where noncoding single nucleotide variants (SNVs) are associated with both T2D and visceral adiposity (a pathologic endophenotype). We integrated CRISPR targeting of SNV-containing regions and unbiased CRISPR interference (CRISPRi) screening to establish candidate *cis-*regulatory regions, complemented by genetic loss of function in murine diet-induced obesity or ex vivo adipogenesis assays. Nomination of a single causal gene was complicated, however, because targeting of multiple genes near *UBE2E2* attenuated adipogenesis in vitro; CRISPR excision of SNV-containing noncoding regions and a CRISPRi regulatory screen across the locus suggested concomitant regulation of *UBE2E2*, the more distant *UBE2E1*, and other neighborhood genes; and compound heterozygous loss of function of both *Ube2e2* and *Ube2e1* better replicated pathological adiposity and metabolic phenotypes compared with homozygous loss of either gene in isolation. This study advances a model whereby regulatory effects of noncoding variation not only extend beyond the nearest gene but may also drive complex diseases through polygenic regulatory effects.

## Abstract 2

<a></a>This functional genomics study validates non-coding GWAS hits at the UBE2E2 locus as determinant for adiposity and type 2 diabetes traits, but unexpectedly through polygenic effectors.

## Introduction

Type 2 diabetes mellitus (T2D) is driven in part by genetic risk with estimates of heritability based on family and twin studies of up to approximately 70% (1). Importantly, T2D phenotypes arise due to single gene variants in a minority of patients (2). By contrast, T2D genome-wide association studies (GWAS) have collectively identified several hundred putative disease–related genes that contribute to the polygenic architecture of T2D heritability (3). The complexity of T2D genetics is compounded by diverse pathological mechanisms involving multiple different metabolically active tissues, including pancreas, liver, skeletal muscle, and adipose tissue, among others. GWAS of relevant tissue specific endophenotypes further expands the list of candidate metabolic risk genes, while focusing mechanistic studies on specific cell and tissue types.

The link between obesity and T2D provides rationale to understand how adipose tissue endophenotypes may contribute to T2D (4, 5). Indeed, various traits related to adipose tissue quantity, distribution, or quality predict cardiometabolic disease risk, including body fat percentage (6), waist circumference (7, 8), visceral adiposity (9), and adipose tissue cellularity (10). Moreover, GWAS have identified candidate genes for relevant adiposity phenotypes that are measurable with imaging modalities such as CT (11). This provided rationale for a prior GWAS meta-analysis of pathogenic adiposity phenotypes, which identified — among other candidate genes — E2 conjugating enzyme E2 (*UBE2E2*) (12). The *UBE2E2* locus is of particular interest due to its identification by independent GWAS for T2D (13), providing rationale for the hypothesis that *UBE2E2* is a causal gene for T2D that is operative through its role in adipose tissue development or function.

T2D and visceral adiposity single nucleotide variants (SNVs) at the *UBE2E2* locus are in noncoding regions of the genome, and therefore, the initial identification of *UBE2E2* as a candidate gene was largely driven by the “nearest gene” assumption (12, 13). However, reductionist functional genomics studies demonstrate numerous examples where phenotypes are driven by causal genes that are regulated by variation in distant noncoding regions (14, 15). Moreover, predictive models suggest that a gene distal to the nearest gene is causal for roughly half of GWAS signals (16, 17), underscoring the importance of in-depth functional assessment of candidate genes, particularly those nominated by associations with noncoding variants.

In our prior work, the dynamic expression of *Ube2e2* in murine adipose tissue coupled with attenuation of adipocyte development with *Ube2e2* loss of function in cultured murine adipocyte progenitor (AP) cells advanced *UBE2E2* as the causal gene for adiposity phenotypes (12). In this study, we sought to further interrogate the nearest gene assumption to explain phenotypic associations for noncoding SNVs at the *UBE2E2* locus. We integrated functional genomics approaches in adipocyte precursor cells with in vivo murine genetic loss-of-function studies, establishing support for *UBE2E2* as a causal contributor to adiposity phenotypes; however, our functional genomics assays also provided evidence of one or more additional causal genes in the *UBE2E2* neighborhood, including the gene coding the related UBE2E1. Collectively, these data establish the importance of the *UBE2E2* locus to metabolic homeostasis. Through identification of more than 1 contributing “causal gene” to adiposity and T2D phenotypes for variants near the *UBE2E2* locus, this work provides experimental evidence for extension of the polygenic disease paradigm to include consideration of disease-associated variants and their gene neighborhoods.

## Results

### UBE2E2 loss of function in mice partially phenocopies human GWAS prediction.

We previously identified UBE2E2 as a regulator of adipocyte differentiation using shRNA-mediated loss of function in an ex vivo adipogenesis assay with primary murine AP cells (12). When coupled with human GWAS demonstrating associations between SNVs at the *UBE2E2* locus T2D and with visceral — as opposed to s.c. — adiposity, we formulated the hypothesis that UBE2E2 loss of function drives negative metabolic sequelae by impairing adipogenesis and healthy adipose tissue development in metabolically protective s.c. depots — i.e., that UBE2E2 loss of function would lead to a phenotype on the lipodystrophic spectrum.

**Figure 1:**
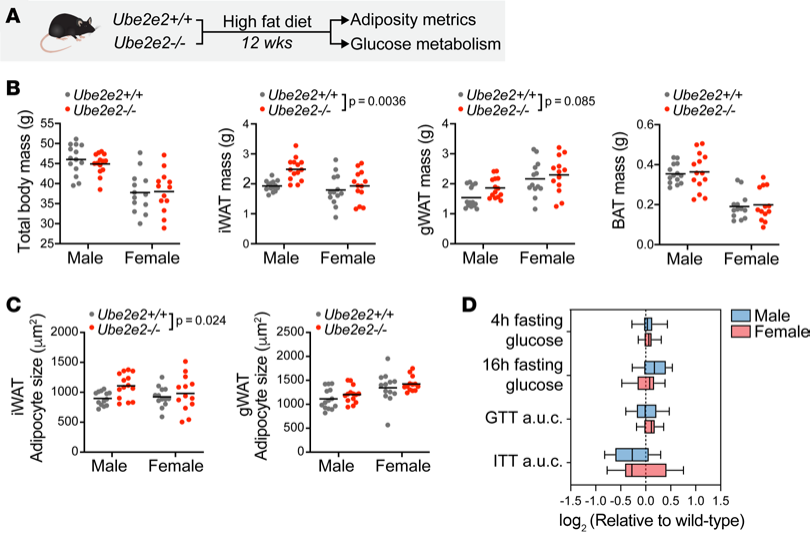
Increased adiposity in mice with *Ube2e2* loss of function. (**A**) Schematic depicting characterization of *Ube2e2^–/–^* mice, relative to WT controls. Ten-week-old mice were subjected to metabolic stress of diet-induced obesity. Metrics of adiposity and glucose metabolism were assessed after 12 weeks of high-fat feeding. (**B**) Total body mass and adipose tissue masses measured after 12 weeks of high-fat diet. Significance was assessed by 2-way ANOVA with *P* values reported for genotype effect inclusive of both sexes. Line indicates mean. *n* = 13–14. (**C**) Adipocyte size indicated by mean cross-sectional for each mouse depot. Significance assessed by 2-way ANOVA with *P* values reported for genotype effect inclusive of both sexes. Line indicates mean. *n* = 13–14. (**D**) Metrics of glucose metabolism assessed after 12 weeks of high-fat feeding, indicating no significant difference relative to WT mice. Box plots show median, with interquartile range and whiskers indicating maximum and minimum. *n* = 13–14.

To investigate the functional importance of UBE2E2, in vivo, we generated *Ube2e2^–/–^* mice using a CRISPR approach (Figure 1A and Supplemental Figure 1; supplemental material available online with this article; https://doi.org/10.1172/jci.insight.184140DS1). We first tested *Ube2e2* loss of function in mice with an ex vivo adipogenesis assay. In this manuscript, we utilized different cellular adipogenesis models, guided by specific experimental goals and practical considerations: (a) isolation of small populations of primary AP cells for characterization of genetic mouse models such as *Ube2e2^–/–^* mice; (b) a human mesenchymal stem cell line for experiments in which a human genome was critical; and (c) murine 3T3L1 preadipocytes for parallel functional loss-of-function analyses of panels of genes, given their high adipogenic efficiency and amenability to viral transgenesis. Indeed, ex vivo analysis of primary APs from *Ube2e2^–/–^* mice provided a first indication of functional consequences to UBE2E2 loss of function, as progenitors isolated from the KO strain demonstrated attenuated adipogenesis relative to cells isolated from WT mice (Supplemental Figure 1), consistent with previously observed attenuation of adipogenesis in primary AP cells when *Ube2e2* was targeted short hairpin RNA (shRNA) (12).

We next examined whether the *Ube2e2^–/–^* mice would recapitulate adiposity phenotypes, using the diet-induced obesity model. We did not observe a gross difference in body weight (BW) in adult mice after 12 weeks of high-fat feeding (Figures 1B and Supplemental Figure 1). However, *Ube2e2^–/–^* mice exhibited increased inguinal fat mass and a trend toward increased gonadal fat mass, without a difference in classical brown adipose tissue. Consistent with the increase in adiposity, we also detected increased adipocyte size (Figure 1C). Given T2D SNV near *UBE2E2*, we tested for impaired metrics of glucose homeostasis in diet-induced obese *Ube2e2^–/–^* mice. We measured glucose after 4 and 16 hours of fasting, detecting no difference between WT and *Ube2e2^–/–^* mice (Figure 1D). Similarly, we found no significant difference in glucose or insulin tolerance testing between WT and *Ube2e2^–/–^* mice (Figures 1D and Supplemental Figure 1). Collectively, these data suggest that UBE2E2 loss of function in mice results in modestly increased adiposity without overt obesity and without an obvious disruption of glucose homeostasis. Unlike the human GWAS, which suggested increased visceral fat relative to s.c. fat, in *Ube2e2^–/–^* mice, we observed a directionally consistent effect in the 2 white adipose depots with only the s.c. inguinal depot reaching statistical significance. While these data are consistent with an adiposity phenotype, they do not suggest a lipodystrophic phenotype as we had hypothesized.

### Identification of multiple candidate causal genes in the topological neighborhood of UBE2E2.

**Figure 2:**
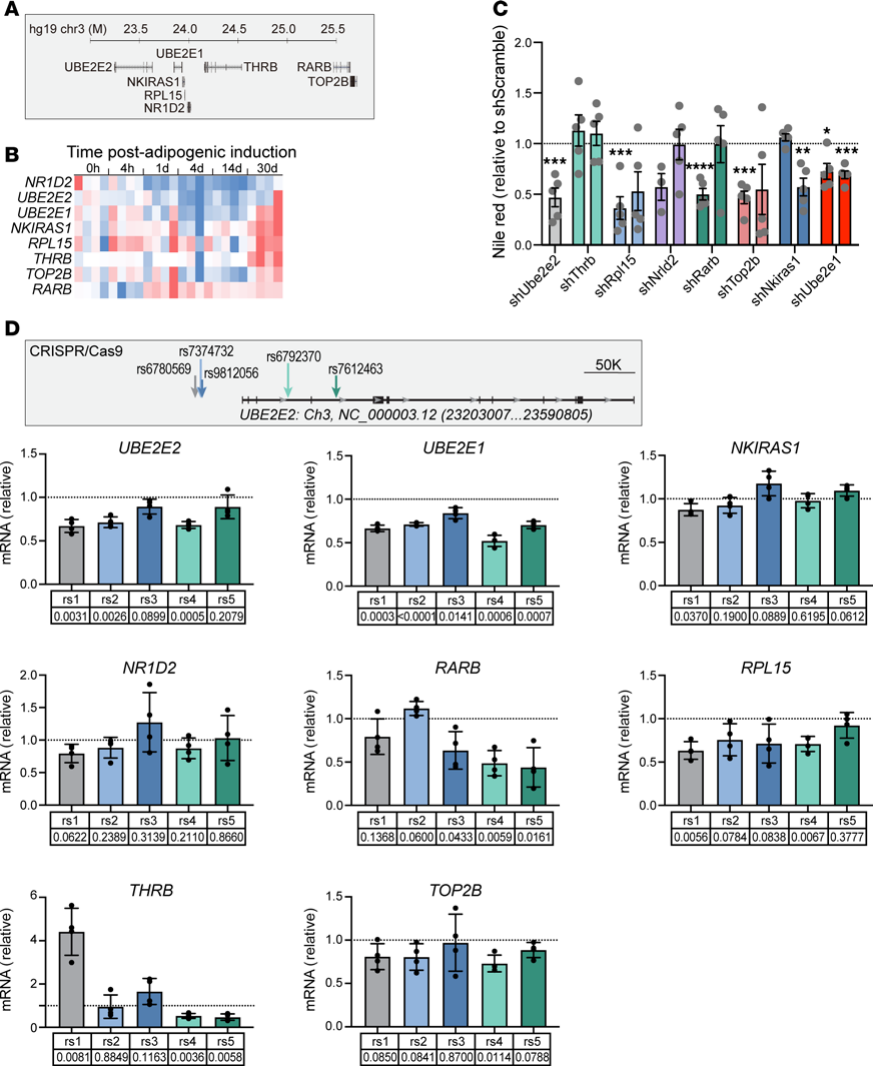
Identification of multiple candidate causal genes in the topological neighborhood of *UBE2E2*. (**A**) UBE2E2 neighborhood gene map. (**B**) Heatmap showing relative changes in expression of neighborhood genes by qPCR during adipogenic differentiation of mesenchymal stem cells (MSC) in vitro. (**C**) Adipogenesis assay with shRNA knockdown of neighborhood genes normalized to sh-scramble control (dotted line at 1.0) with *Ube2e2* knockdown shown as positive control. Each dot represents the mean of technical replicates for an individual biological replicate experiment (*n* = 5, except Nrld2a, which is *n* = 3). For each gene knockdown, significance was assessed by 1-sample *t* test relative to null expectation equal to scramble control value of 1: *P < 0.05; **P < 0.01; ***P < 0.005; ****P < 0.001. (**D**) CRISPR/Cas9 excision of ~200 bp SNV containing regions in cultured MSC. Expression of genes in the *UBE2E2* topological neighborhood was assessed by qPCR, with each dot indicating mean of technical replicates for an independent biological replicate experiment. To merge different biological replicate experiments (*n* = 4), data were expressed relative to control and *P* values were calculated by 1-sample *t* test relative to expected null value = 1.

Our initial identification of *UBE2E2* as a candidate gene regulating visceral adiposity relied on a nearest gene assumption as the lead SNV resides in noncoding DNA (12). Distinct SNVs linked to T2D and not in linkage disequilibrium with our lead SNV are also in noncoding DNA. Given only partial recapitulation of the phenotypes predicted by GWAS with UBE2E2 loss of function in mice, we revisited our hypothesis of *UBE2E2* as the causal gene. We first applied functional assays, previously applied in the prioritization of *UBE2E2* (12), but broadened the analysis to include additional genes in the topological neighborhood, as defined by residing within 500 kb of the *UBE2E2* locus or within the predicted *UBE2E2* topologically associating domain (TAD) (Figure 2A). This included *UBE2E2* and *UBE2E1 —* both of which have supporting expression quantitative trait locus (eQTL) evidence for SNV regulatory effects, albeit not consistently in adipose tissue itself — and additional genes without known SNV associated eQTL (GTEx database V10). Although we cannot exclude the possibility of non-*UBE2E2* causal genes located within or beyond the TAD, we reasoned that this approach would capture genes most likely affected by *UBE2E2-*associated SNV. In mesenchymal stem cells (MSC) directed to adipocyte differentiation, we observed dynamic changes in *UBE2E2* expression by quantitative PCR (qPCR), as well as other genes in the topological neighborhood (Figures 2B and Supplemental Figure 2). With shRNA knockdown of genes in the topological neighborhood in murine 3T3L1 cells, we found reproducible attenuation of adipogenesis when targeting *Ube2e1* with 2 different shRNAs (Figure 2C). For several other neighborhood genes, 1 out of 2 shRNAs attenuated adipogenesis, including *Nkiras1*, *Rpl15*, *Rarb*, and *Top2b*. Such differences could not be easily explained by correlative differences in estimated knockdown efficiency (Supplemental Table 1). Though we do not exclude an adipogenic function for genes where there was a discrepant result between 2 targeting hairpins, we advance *Ube2e1* as a candidate adipogenic factor with higher confidence given that both targeting hairpins had a functional effect.

In the absence of a single clear functionally important gene, we next tested whether CRISPR/Cas9 targeting of the noncoding regions containing adiposity/T2D SNVs near *UBE2E2* would modulate its expression or expression of other genes in the topological neighborhood. We delivered CRISPR/Cas9 and guide RNA (gRNA) flanking approximate 100–200 bp regions, inclusive of the lead SNVs, to hMSC (Figure 2D). We performed 4 independent biological replicate experiments and assessed the modulation of *UBE2E2* and additional genes contained within the topological neighborhood by qPCR (Figure 2D). Targeting of the noncoding region containing the lead SNV from the GWAS metaanalysis that identified *UBE2E2* as a candidate gene for visceral adiposity led to a significant reduction (approximately 25%) in *UBE2E2* expression. Overall, targeting 3 of 5 SNV regions resulted in statistically significant reductions in *UBE2E2* expression. When we considered additional genes contained within the topological neighborhood, we discovered a more consistent effect on *UBE2E1*, with significant reduction in expression with targeting each of the 5 SNV-containing regions. The expression of 2 neighborhood genes, NR1D1 and NKIRAS1, were not modified by editing any of the 5 SNV regions. While most edits led to reduced expression, excision of a region containing rs6780569 resulted in an approximate 4-fold increase in expression of *THRB*. These data collectively suggest that multiple genes in the topological neighborhood of *UBE2E2* display evidence of potential functionality, including *UBE2E1*, which met multiple criteria as a second potential candidate causal gene (Table 1).

### Overlap of putative cis-regulators of UBE2E2 and UBE2E1.

**Figure 3:**
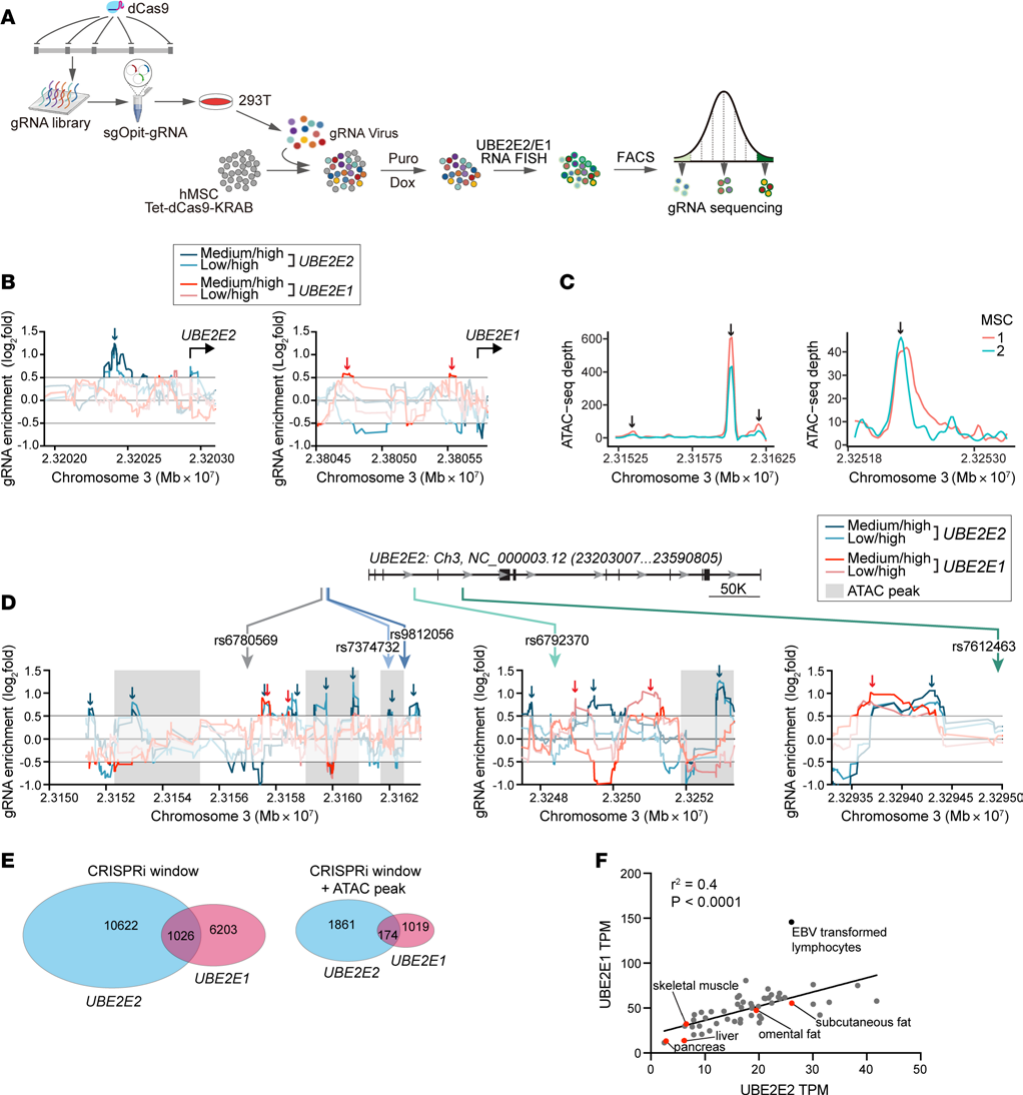
Integration of ATAC-Seq and CRISPRi screen to define *cis*-regulatory landscape at the *UBE2E2* locus. (**A**) Schematic depicting CRISPRi protocol to identify candidate *cis*-regulatory domains for *UBE2E2* and *UBE2E1* expression as quantified by RNA FISH and flow cytometry in hMSC. (**B**) Graphs of log_2_fold change in gRNA enrichment as a function of sliding window location on chromosome 3 at the putative *UBE2E2* and *UBE2E1* promoters. (**C**) ATAC-Seq tracks of hMSC showing peaks (arrows) near GWAS SNV and shown by gray shading in **D**. (**D**) gRNA enrichment in the vicinity of the 5 GWAS SNV for adiposity and T2D traits. (**E**) Venn diagrams showing partial overlap of candidate *UBE2E2* and *UBE2E1* regulatory domains using 2 different levels of stringency: left, candidate domains identified by CRISPRi; right, candidate domains identified by both CRISPRi and ATAC-Seq. (**F**) GTEX data extracted for both *UBE2E2* and *UBE2E1* showing correlation of the respective transcript levels across tissues.

Given that *UBE2E1* was most consistently implicated as a second potential causal gene (Table 1), we next investigated potential polygenic effects of noncoding variation at the *UBE2E2* locus involving both *UBE2E2* and *UBE2E1*. We integrated ATAC-Seq analyses of hMSC with CRISPR interference (CRISPRi) screening to identify potential regulatory regions controlling *UBE2E2* expression and/or *UBE2E1* expression. For the CRISPRi screen, we utilized in situ RNA probes to report on *UBE2E2* or *UBE2E1* expression in hMSC coupled with flow cytometry as a transcriptional readout as demonstrated by others (18, 19) (Figure 3A). This entailed introduction of a custom gRNA library by viral transgenesis covering a region of hg19.Chr3: 22,500–24,500 kb, inclusive of all known SNVs associated with the *UBE2E2* and *UBE2E1* genes and all known regulatory regions within a single TAD across different cell types (20). MSC were sorted by flow cytometry to select and sequence cell populations that corresponded to high or low *UBE2E2* or *UBE2E1* expression.

We applied a sliding window analysis of consecutive gRNA to identify candidate regulatory domains. Principal component analysis (PCA) demonstrated discrimination of different cell populations, with clearest distinction of *UBE2E2^lo^* and *UBE2E2^med^* and *UBE2E1^hi^* and *UBE2E1^med^* from the respective high-expressing populations (Supplemental Figure 3). We performed pairwise comparisons of gRNA enrichment focusing on differential gRNA enrichment between either of the 2 low-expressing populations relative to the high-expressing population. By contrast to what has previously been shown when this method has been used to identify potent *cis-*regulatory domains at loci that are hyperexpressed, such as *MYC* in cancer cells, hotspots of gRNA enrichment were modest in scale, which is consistent with similarly modest effects on gene expression with CRISPR excision of SNV-containing regions. For example, when we focused specifically on gRNA-targeting regions in proximity to putative promoters within 1,000 bp of the transcriptional start site (TSS) upstream of exon 1 (Figure 3B), we observed enrichment of gRNA in the suppressed populations with log_2_-fold differences in the range of 0.5–1. While the on-target action of CRISPRi results in recruitment of a repressive complex to putative *cis*-regulatory domains, it has previously been noted that additional regulatory mechanisms may also be operative, including paradoxical augmentation of gene expression through sequestration of repressive factors. Consistent with this dichotomy, we found evidence of differential gRNA enrichment consistent with both mechanisms; for example, in the vicinity of the *UBE2E1* TSS, we observed enrichment of gRNA in *UBE2E1^lo^* cells and in *UBE2E2^hi^* cells (Figure 3B).

We next examined genomic regions in the vicinity of the 5 GWAS SNVs (Figure 3, C and D). In each case, different patterns became evident with respect to the SNV proximity to ATAC peaks or gRNA hotspots. Two of the SNVs (rs7374732, rs9812056) mapped to an ATAC peak, 2 were within 5 kb of an ATAC peak (rs6780569, rs6792370), and 1 was approximately 17 kb from the nearest ATAC peak (rs7612463) (Figure 3, C and D, and Table 2). Each of the SNVs mapped near 1 or more candidate regulatory domains identified by the CRISPRi screens for both *UBE2E2* and *UBE2E1*, with 1 prominent example of closely overlapping signals of gRNA enrichment in low expressing cells near rs7612463 (Figure 3D). We next leveraged these data to examine the degree of potential overlap of candidate *UBE2E2* and *UBE2E1* regulatory domains. We applied a permissive filter of a nominal *P* < 0.05 for pairwise comparisons and then secondarily limited the analysis to gRNA enrichment signals consistent with an on-target CRISPRi effect (gRNA enrichment in cells with lower gene expression) or candidate regulatory domains that correlated with ATAC-Seq peaks. At each level of stringency, we found a subset of candidate *cis*-regulatory domains common to both *UBE2E2* and *UBE2E1* (Figure 3E). If there was coregulation of *UBE2E2* and *UBE2E1*, then we predicted that expression of these 2 genes might be correlated. To test this, we interrogated the GTEX database and assessed for a correlation at the transcriptomic level across cells/tissues (Figure 3F). Indeed, we discovered a strong positive correlation (*R*^2^ = 0.4, *P* < 0.0001). Collectively, these data are consistent with a model whereby noncoding variants at the UBE2E2 locus may regulate both the nearest gene (*UBE2E2*) and the more distant *UBE2E1*.

### Functional divergence of UBE2E2 and UBE2E1.

**Figure 4:**
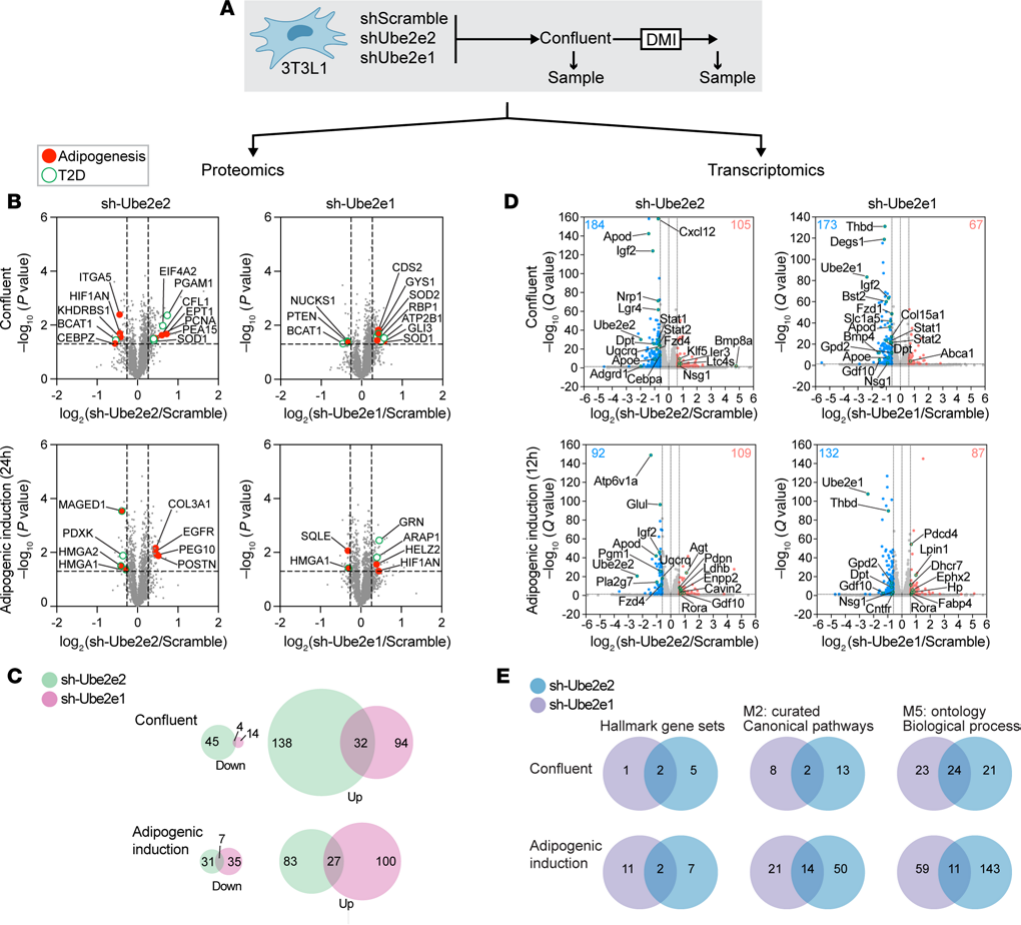
Functional divergence of UBE2E2 and UBE2E1. (**A**) Schematic depicting multi-omics investigation of molecular programs with *Ube2e2* and *Ube2e1* loss of function. 3T3L1 cells expressing shUbe2e2, shUbe2e1, or scramble control were analyzed at confluency or after adipogenic induction (DMI). (**B**) Volcano plots demonstrate differential protein levels in cell homogenates. Proteins previously linked to adipogenesis or T2D are indicated. (**C**) Venn diagrams showing overlap of differentially regulated proteins. (**D**) RNA-Seq data visualized by volcano plot. (**E**) Venn diagrams showing overlap of differentially regulated gene programs as assessed by GSEA.

UBE2E2 and UBE2E1 are both members of the same family of E2 conjugating enzymes, raising the question of whether they are functionally redundant, particularly given that genetic targeting of both genes inhibited adipogenesis, in vitro. Moreover, neither member has been extensively studied; therefore, the current literature does not provide a clear indication of shared protein targets or E3 ligase cooperativity. To explore this question, we sought to test for molecular redundancy. We transduced 3T3L1 cells with previously validated shRNA plasmids targeting *Ube2e2* or *Ube2e1* and confirmed inhibition of adipogenesis using Oil Red O staining and qPCR for adipogenic genes as complementary readouts to the fluorescence-based screen shown in Figure 2C (Supplemental Figure 4). We then performed comparative tandem mass tag–based (TMT-based) quantitative proteomics on confluent 3T3L1 cells and again 24 hours after adipogenic induction (Figure 4A). At each time point, differential proteins included products of genes previously demonstrated to regulate adipogenesis or linked to T2D (Figure 4B). There were generally a greater number of proteins that increased with targeting of either *Ube2e2* or *Ube2e1*, consistent with the canonical role of E2 conjugating enzymes in proteosome-mediated degradation (Figure 4C). Although a subset of differential proteins was shared between *Ube2e2-* and *Ube2e1-*knockdown cells at each time point, the majority were distinct.

We also performed RNA-Seq in cells under similar culture conditions to determine if the proteomic level differences would translate into different gene programs (Figures 4D). We performed gene set enrichment analysis (GSEA) to test the degree to which differentially regulated pathways were shared. We included hallmark gene sets (MH; normalized *P* < 0.05), curated canonical pathways (M2, normalized *P* < 0.01), and Biological Process ontology (M5, normalized *P* < 0.01) gene sets from the GSEA Mouse MSigDB Collections and found limited overlap between *Ube2e2-* and *Ube2e1*-knockdown cells, regardless of adipogenic induction (Figure 4E and Supplemental Figure 4). This unbiased multiomics characterization of the UBE2E2- and UBE2E1-dependent molecular programs in 3T3L1 preadipocytes links the regulatory functions of these 2 proteins to pathways of relevance to adipocyte development and/or function, while arguing against molecular redundancy.

### UBE2E1 loss of function disrupts glucose homeostasis in mice.

**Figure 5:**
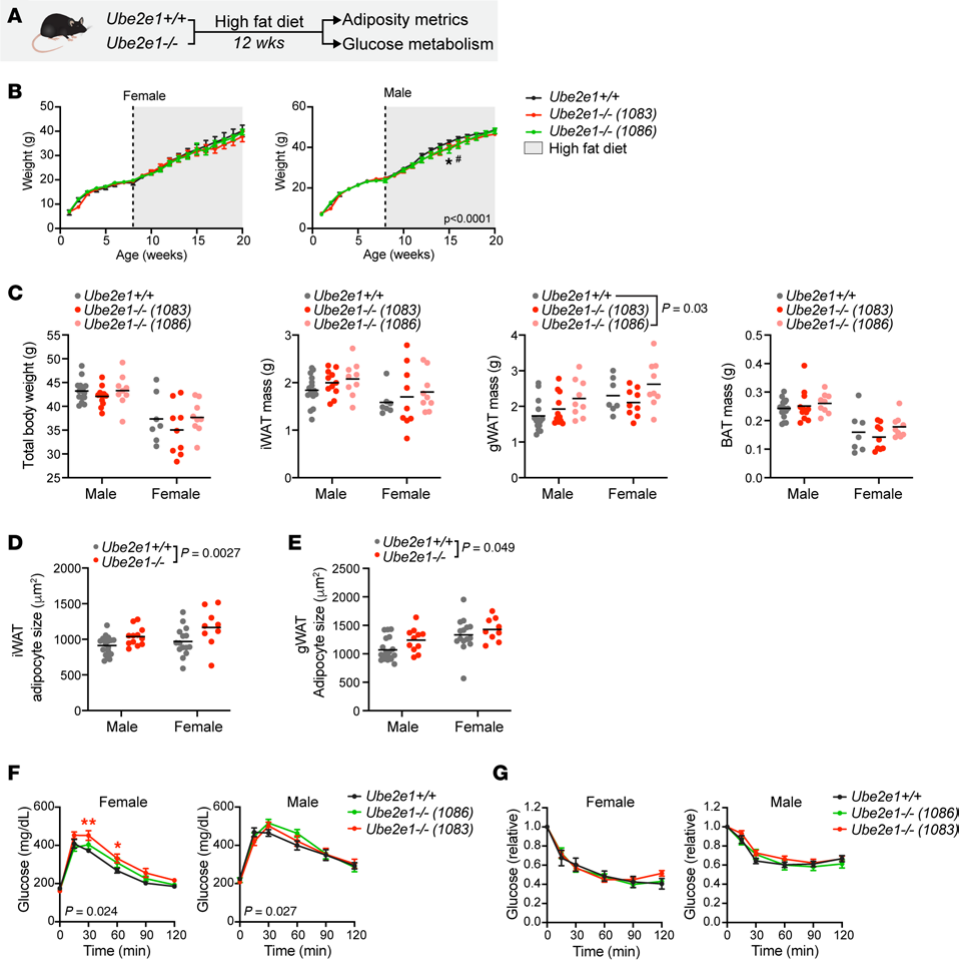
UBE2E1 loss of function in mice impairs glucose metabolism with diet-induced obesity. (**A**) Schematic depicting experimental protocol. Eight-week-old mice were entered into the study. Female, *n* = 8–9; male, *n* = 10–15. (**B**) Body weight evolution with diet-induced obesity in *Ube2e1*-KO mice. Female mice showed no difference in body weight over time. Curves for the 2 *Ube2e1* mutant lines diverged from control in male mice but converged to control by the end of the 12-week high-fat feeding period. Left: male mice; right: female mice. No. 1086, ^#^*P* < 0.05; No. 1083, *P < 0.05. (**C**) Terminal body mass and adipose tissue weights at time of sacrifice after 12 weeks of diet-induced obesity. Reported *P* values for 2-way ANOVA; genotype effect is inclusive of both sexes. (**D**) Adipocyte size indicated by mean cross-sectional area for inguinal white adipose tissue (iWAT). Significance assessed by 2-way ANOVA with *P* value reported for genotype effect. Line indicates mean. (**E**) Adipocyte size as in **D** for gonadal white adipose tissue (gWAT). Significance assessed by 2-way ANOVA with *P* value reported for genotype effect. Line indicates mean. (**F**) GTT of mice in **B** after 12 weeks of high-fat feeding. Two-way ANOVA with *P* value for genotype × time effect reported in the bottom left corner. After Dunnett’s adjustment: * 1083 *P* < 0.05; ** 1083 *P* < 0.005. (**G**) ITT of mice in “b” after 12 weeks high fat feeding.

Given the collective data suggesting *UBE2E1* as an alternative candidate causal gene for GWAS SNV near *UBE2E2*, we next explored whether genetic loss of UBE2E1 would drive adiposity and metabolic phenotypes. We employed CRISPR/Cas9 to target *Ube2e1*, generating 2 lines that exhibited disruption of the target exon by sequencing (Supplemental Figure 5). Like *Ube2e2-*null mice, primary AP cells isolated from *Ube2e1-*null mice exhibited an adipogenesis defect ex vivo (Supplemental Figure 5). In an early experiment, we also discovered evidence of impaired glucose tolerance after 12 weeks of high-fat feeding (Supplemental Figure 5). This motivated a second, better-powered diet-induced obesity experiment (Figure 5A). Over 12 weeks of high-fat feeding, BW of WT and *Ube2e1-*null mice remained similar except for a transient period when the weights of male *Ube2e1^–/–^* mice lagged WT controls, an effect that was no longer evident at completion of the 12-week feeding period (Figure 5B). In contrast to *Ube2e2-*null mice (Figure 1B), *Ube2e1^–/–^* mice had a less overt adiposity phenotype with a significant difference in fat mass only observed in the gonadal depot of 1 of the 2 KO lines (Figure 5C), although there was an increase in adipocyte size with Ube2e1 loss of function (Figure 5, D and E). In presacrifice glucose-tolerance testing (Figure 5F), we observed impaired glucose tolerance that was directionally consistent in both sexes and in line with the initial pilot experiment (Supplemental Figure 5), yet it was more robustly evident in female mice (Figure 5F). In neither sex did we observe a difference in insulin tolerance (Figure 5G). Collectively, these data point to mild disruption in glucose homeostasis with global *Ube2e1* targeting.

### Compound heterozygous loss of Ube2e2 and Ube2e1 recapitulates GWAS phenotypes.

**Figure 6:**
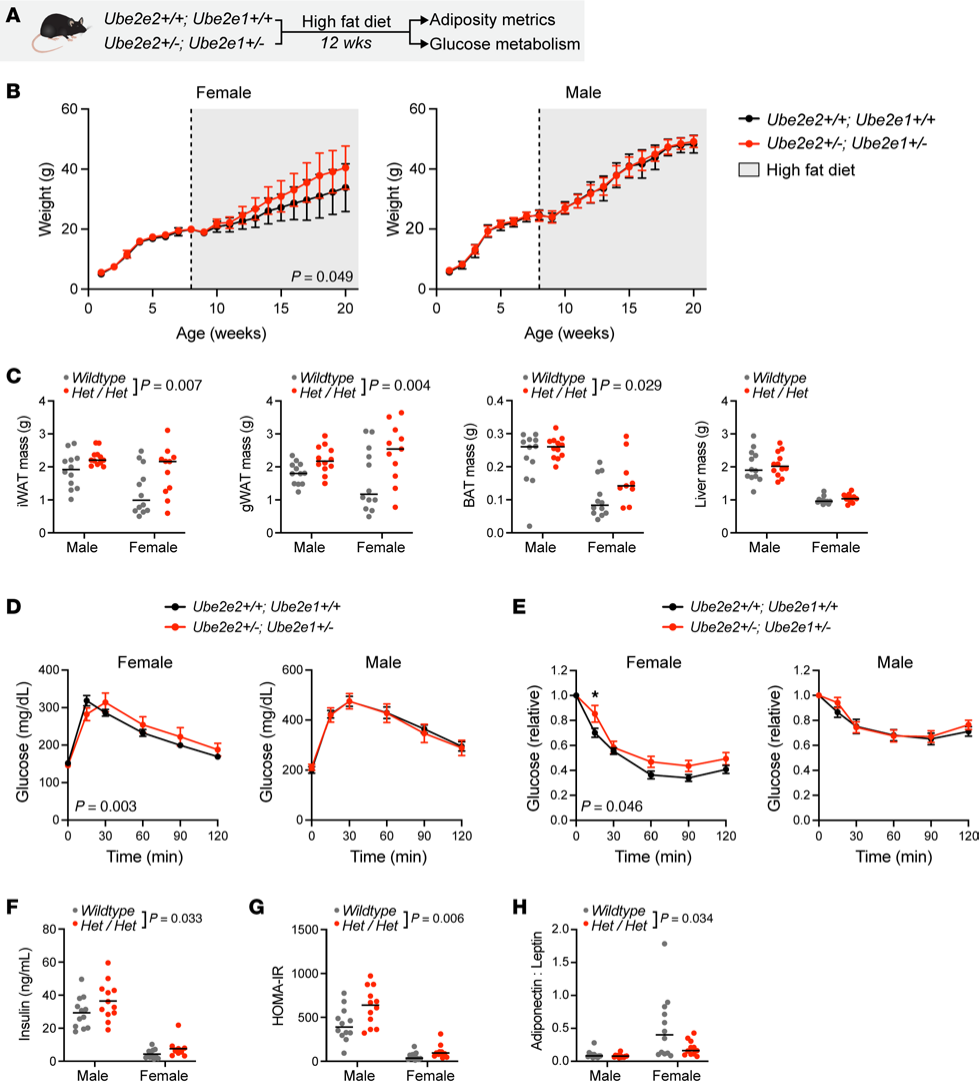
Augmented adiposity and dysregulated glucose homeostasis with compound heterozygous loss of *Ube2e2* and *Ube2e1*. (**A**) Schematic depicting experimental protocol. Eight-week-old mice were entered into the study. (**B**) Body weight evolution with diet induced obesity (*n* = 11–13). *P* value for genotype effect in 2-way ANOVA. (**C**) Terminal adipose tissue and liver weights at time of sacrifice after 12 weeks of diet-induced obesity. Reported *P* values for 2-way ANOVA, genotype effect inclusive of both sexes (*n* = 9–12). (**D**) GTT after 12 weeks of high-fat feeding. Two-way ANOVA with *P* value for genotype × time effect. (**E**) ITT after 12 weeks of high-fat feeding. Two-way ANOVA with *P* value for genotype × time effect reported in the bottom left corner (*n* = 11–12). After Šidák adjustment: *P < 0.05. (**F**) Terminal serum insulin levels. Reported *P* value for 2-way ANOVA, genotype effect (*n* = 11–12). (**G**) Terminal HOMA-IR. Reported *P* value for 2-way ANOVA, genotype effect (*n* = 11–12). (**H**) Attenuation of adipose tissue function as assessed by terminal serum adiponectin:leptin ratio. Reported *P* value for 2-way ANOVA, genotype effect (*n* = 11–12). Sensitivity analysis performed by removing WT female outlier with resultant *P* = 0.031.

No single genetic manipulation in our functional genomics analyses replicated the full range of predicted adipose and metabolic outcomes. If UBE2E2 and UBE2E1 do indeed regulate distinct molecular programs, however, targeting both genes might be necessary to fully recapitulate metabolic phenotypes. Moreover, the total loss-of-function model is extreme relative to the modest effects on gene expression observed with targeting of noncoding regions near *UBE2E2*. Therefore, we reasoned that compound heterozygous loss of function of *Ube2e2* and *Ube2e1* would more closely reflect the observed effects of targeting putative regulatory domains in the noncoding regions around *Ube2e2*. Therefore, we subjected *Ube2e2^+/–^ Ube2e1^+/–^* and *Ube2e2^+/+^ Ube2e1^+/+^* control mice to diet-induced obesity (Figure 6A). Male mice exhibited similar BW trajectories with high-fat diet, whereas female compound heterozygous mutant mice gained more weight than WT controls (Figure 6B). Compound heterozygous loss of *Ube2e2* and *Ube2e1* also resulted in increased adiposity evident across depots (Figure 6C). Glucose- and insulin-tolerance testing demonstrated modest effects in female mice (Figure 6, D and E). The negative effect on metabolic health was more evident in terminal measurements of insulin and HOMA-IR, where targeting *Ube2e2* and *Ube2e1* augmented both metrics (Figure 6, F and G). Interestingly, despite no difference in BW between male control and mutant mice, insulin and HOMA-IR measures were directionally consistent with the female mice that did exhibit an overt BW phenotype. Nonetheless, sex may be an additional modifying factor of importance even though this study did not perform the additional metabolic phenotyping (e.g., metabolic cage studies) that might reveal candidate mechanisms for the modifying effect of sex on BW gain.

The ratio of serum adiponectin to leptin is an emerging metric of adipose tissue function (21, 22). We measured each adipokine at the time of sacrifice, finding a reduction in this ratio in compound heterozygous mice consistent with adipose tissue dysfunction (Figure 6H and Supplemental Figure 5). Therefore, these collective data demonstrate that targeting *Ube2e2* and *Ube2e1* with partial loss of function — as might be observed with disruption of a noncoding domain regulating both genes — is sufficient to drive increased adiposity, adipose tissue dysfunction, and diabetic pathophenotypes.

## Discussion

In this study, we performed in vitro and in vivo functional genomics analyses of the *UBE2E2* locus previously linked to adiposity and T2D traits. Irrespective of the assay, nomination of a single causal gene was complicated because: (a) targeting of multiple genes in the neighborhood of *UBE2E2* attenuated adipogenesis in an in vitro functional assay; (b) CRISPR excision of SNV-containing noncoding regions and a CRISPRi regulatory screen across the locus suggested concomitant regulation of *UBE2E2*, the more distant *UBE2E1*, and perhaps additional genes; (c) *Ube2e2* and *Ube2e1* genetic loss of function in mice independently resulted in pathophenotypes resembling those predicted by GWAS signals; and (d) compound heterozygous loss of function of both *Ube2e2* and *Ube2e1* better phenocopied the collective adiposity and metabolic phenotypes than homozygous loss of either gene in isolation. Our collective data implicates a 2-part model: (a) that a subset of noncoding variants affect expression of more than 1 gene in the topological neighborhood of *UBE2E2* and (b) that the interrelated phenotypic effects on adiposity and glucose homeostasis are driven by this polygenic regulatory effect. Our study provides an example of the increasingly recognized phenomenon of regulatory effects of noncoding variation extending beyond the nearest gene, while also implicating polygenic modulation of relevant metabolic phenotypes by noncoding variation.

The fact that *UBE2E2* and *UBE2E1* both code for members of the E2 ubiquitin conjugating family of proteins — coupled with attenuation of adipogenesis with UBE2E2/UBE2E1 loss of function — raises the question of functional redundancy. E2-conjugating enzymes transfer activated ubiquitin to proteins, an interaction mediated by E3 ligases, which impart protein target specificity (23). Although the family of > 600 distinct E3 ligases exceeds the number of potential E2 partners by over 1 order of magnitude, E3 ligases may still interact with multiple E2 enzymes (23–26). This combinatorial complexity decreases the likelihood of complete functional redundancy of 2 different E2-conjugating enzymes, consistent with our finding of only partial molecular overlap in comparative analyses of the proteomic and transcriptomic consequences of targeting each protein independently in 3T3L1 cells. Moreover, the murine genetic loss-of-function models did not phenocopy one another; UBE2E2 loss of function increased adiposity without overt metabolic consequences, whereas UBE2E1 loss of function worsened metrics of glucose homeostasis with minimal effects on adiposity.

One premise underpinning this study is that adiposity and T2D traits are interrelated — that adiposity endophenotypes are drivers of T2D — prompting the question of why the augmented adiposity observed in *Ube2e2-*null mice did not translate into detectable metabolic dysfunction. In murine genetic models, metabolic pathophenotypes are often not easily detectable without a secondary challenge, with the diet-induced obesity model used here being a common experimental stressor. It is possible that UBE2E2-dependent metabolic phenotypes might emerge more dramatically in contexts not tested in our study, such as with aging or with alternative metabolic stressors. It is also possible that, in the absence of an overt obesity phenotype, the metabolic consequences of a modest increase in adiposity are not easily detectable with standard mouse phenotyping methods. Increased adiposity is not necessarily deterministic for T2D, given precedent for coexistence of insulin sensitivity with extreme obesity in a small subset of genetic murine models (27, 28). Although we cannot exclude an uncoupling of adiposity and metabolic dysfunction, considering the large body of evidence linking adipose tissue excess and/or dysfunction to metabolic disease coupled with the deleterious metabolic effects of compound *Ube2e2/Ube2e1* heterozygous loss of function, the *Ube2e2*-null phenotype of increased adiposity is more likely to be pathological rather than metabolically benign.

In addition to being a risk factor for cardiometabolic disease, visceral adipose tissue is more permissive to adipogenesis (29, 30), which may explain in part the s.c. to visceral fat redistribution observed with some nongeneralized lipodystrophies — i.e., those that do not completely abolish all white adipocyte development. Based on a GWAS signal for visceral adiposity coupled with an associated in vitro adipogenesis defect, our a priori hypothesis was that *UBE2E2* loss of function would manifest as a lipodystrophic phenotype in mice. It is important to acknowledge, therefore, that we found no evidence of a lipodystrophic phenotype with either *UBE2E2* or *UBE2E1* genetic loss of function, in vivo, as increased adiposity extended to the inguinal s.c. depot. This discrepancy could simply reflect mouse and human differences in the biology of specific fat depots or, alternatively, the additional modifying effects of causal genes beyond *UBE2E2* and *UBE2E1*.

How does this study then reflect the utility of the adipogenesis assay as a functional genomics tool, considering the apparent disconnect between the in vitro adipogenesis defect and the absence of corresponding overt lipodystrophy with in vivo loss of function? In the standard ex vivo adipogenesis protocol, conversion of adipocyte precursor cells to lipid-laden adipocytes is dependent on cellular processes that are not necessarily specific to the molecular differentiation program, including cell cycle activity (mitotic clonal expansion) at the outset, insulin responsive energy uptake, de novo lipogenesis, and finally lipid droplet formation (31–36). It is therefore possible that aspects of the in vitro adipogenesis assay effectively reports on more general aspects of adipose tissue function beyond fate specification and/or on cellular functionalities that are operative in other metabolically relevant tissues. Despite the limitations of using such cellular assays for functional genomics assessments of complex multicellular, multitissue diseases, the adipogenesis assay arguably fulfilled its utility as a screening tool in our study, as both genes that we ultimately nominated for in vivo study manifested relevant loss-of-function phenotypes that can be plausibly linked to the originating GWAS traits. Indeed, other studies have demonstrated predictive power of the adipogenesis assay for relevant metabolic phenotypes or for functional categorization of GWAS genes (37, 38). Therefore, we view the adipogenesis assay as a tool best applied for prioritization — but not definitive discrimination — of candidate disease genes.

The genomics landscape has been reoriented by the recognition of (a) the functional relevance of variation in noncoding DNA, (b) the potential for long-distance *cis*-regulatory effects such that the causal gene for GWAS signals may not be the nearest gene, and (c) the possibility that noncoding regulatory domains may regulate multiple gene targets, although there are limited empirical examples of this third concept (39–47). In establishing functional relevance for the *UBE2E2* locus to metabolic disease, this study also implicates all 3 of these mechanisms underpinning functional manifestations of noncoding variation. By identifying more than 1 contributing causal gene, we provide an experimental example of how the polygenic paradigm may extend down to the level of individual disease-associated variants. In the functional genomics field, a single candidate causal gene is often nominated based on having the strongest effect size in a functional assay and, therefore, this study suggests the importance of considering supporting roles for additional genes with lesser effect sizes in functional genomics screens. Given the known phenomenon of clustering of functionally related genes (48, 49), it is possible that the interplay between noncoding genetic variation and causal gene clusters is more prevalent than appreciated. It will be important to consider and quantify the contributions of secondary variant-gene couplings that may represent an underappreciated component of the genetic architecture of polygenic disease.

## Methods

### Sex as a biological variable.

Our study examined male and female mice. Some of the reported phenotypes were sexually dimorphic with respect to the scope of effect size.

### Murine studies.

Mice were maintained under a 12-hour dark/light cycle at 22°C ± 2°C receiving food and water ad libitum unless specified. The diet-induced obesity model was performed by administering adult mice a high-fat diet or normal chow control diet (Research Diets, D12492 and D12450J, respectively). *Ube2e2-* and *Ube2e1*-KO mice were generated using CRISPR technology by the Transgenic Mouse Core at Harvard Medical School. gRNAs targeting the coding regions of *Ube2e2* and *Ube2e1* are detailed in Supplemental Table 1. To achieve KO, paired gRNAs and Cas9 protein were microinjected into 1-cell embryos with removal of the targeted regions confirmed by sequencing.

Glucose and insulin tolerance testing was performed as previously described (50). Glucose- and insulin-tolerance–tested mice were fasted for 16 and 4 hours, respectively. Glucose (1.5 g/kg BW) or insulin (0.5–1.0 U/kg BW; for obese mice, we used 1.0 U/kg BW, and for lean mice, we used 0.5 U/kg BW) were administered by i.p. injection, and glucose measurements were performed by sampling a few microliters of blood from the tail vein with a glucometer (Bayer Contour or Medline Pro).

### In vitro adipogenesis models.

Three different adipocyte precursor cell types were utilized for adipogenesis assays in this study: primary AP cells isolated from genetic mouse models cellular adipogenesis models, murine 3T3L1 cells for viral genetic loss-of-function studies due to their high adipogenesis efficiency, and hMSC for experiments where the human genome was critical.

For isolation of primary AP cells, mice were sacrificed at approximately 4 weeks old. Adipose tissue depots were minced and digested in an enzyme cocktail, consisting of collagenase D (Roche) and dispase II (ZenBio or Invitrogen), as described previously (50). After centrifugation (4°C, 400*g*, 10 minutes), the pelleted stromal vascular fraction was subjected to negative selection by column-based magnetic-assisted cell sorting (MACS), using monoclonal anti-CD31 MicroBeads (Miltenyi Biotec) and a biotin-conjugated monoclonal anti-lineage cocktail followed by anti-biotin MicroBeads (Miltenyi Biotec) to negatively select endothelial cells and hematopoietic cells, respectively. The targeted lineage^+^CD31^+^ cells were then depleted by retaining them on a MACS Column in the magnetic field of a MACS Separator (Miltenyi Biotec). The unselected cell fraction was eluted through the column and cultured in DMEM-F/12 GlutaMAX medium (Thermo Fisher Scientific) with 10% Premium FBS (Corning) and penicillin-streptomycin (Pen/Strep; Thermo Fisher Scientific).

3T3L1 cells (ZenBio), hTERT immortalized adipose-derived hMSCs (hMSCs, ATCC, SCRC-4000), and primary AP cells were cultured to confluence. AP cells were cultured in DMEM/F-12 GlutaMAX with 10% FBS, while 3T3L1 cells were cultured in DMEM GlutaMAX with 10% FBS. hMSCs were cultured in MSC basal medium (ATCC, PCS-500-030) supplemented with the MSC growth kit (ATCC, PCS-500-040) according to the manufacturer’s instructions. To stimulate adipogenesis in 3T3L1 cells and primary APs, an adipogenic cocktail containing dexamethasone (1 μM), insulin (10 μg/mL), and isobutylmethylxanthine (0.5 mM) was used. For the induction of hMSCs, rosiglitazone (0.5 μM) was added to the adipogenic cocktail. After 96 hours of induction, the cells were switched to a maintenance medium, which consisted of standard culture medium supplemented with insulin (10 μg/μL). The maintenance medium was refreshed every 2 days. For Nile Red staining, cells were plated in Corning 96-Well Clear Bottom Black plates and with fluorescent signal after adipogenic differentiation quantified with a SpectraMax M5 plate reader (Molecular Devices).

### CRISPR deletion.

For CRISPR deletion experiments in hMSC, we designed gRNAs using the CRISPOR tool to target specific noncoding regions associated with SNVs at the UBE2E2 locus. Two gRNAs (located upstream and downstream of the targeted SNV site, respectively) for each region were selected based on predicted high on-target efficiency and minimal off-target potential. We cloned 2 gRNA into the LentiCRISPR-V2 backbone to generate a dual-gRNA CRISPR lentivirus. After CRISPR-mediated excision, we confirmed the deletion of the target regions by amplifying the regions surrounding the cut sites using PCR. Since 80–290 bp regions were targeted, we were able to confirm the removal of the genomic DNA in this region using genomic DNA PCR.

We assessed the effect of these deletions on the expression of genes located in the neighborhood of the *UBE2E2* locus. Our a priori criteria to define the *UBE2E2* neighborhood included genes located within approximately 500 kb of the *UBE2E2* locus or more distant genes within the putative topological neighborhood identified by incorporating the physical proximity of genomic elements and the regulatory architecture. Specifically, we used chromatin interaction datasets (Hi-C) to identify chromatin loops that define the boundaries of the TAD, which included *UBE2E2*, and within which genes and regulatory elements are more likely to interact (20). We also incorporated chromatin interaction maps, such as promoter capture Hi-C, to detect long-range enhancer-promoter interactions with an emphasis on regulatory elements that may interact with UBE2E2 and overlayed epigenomic data that included all known regulatory effector-binding regions (e.g., histone modification marks such as H3K27ac, H3K4me1, H3K4Me3) and DNaseI hypersensitive (DHS) elements (51).

### CRISPRi screen.

We used cultured hMSC to conduct a parallel CRISPRi screen across the *UBE2E2* and *UBE2E1* loci for regulation of *UBE2E2* and *UBE2E1* expression, following Fulco’s protocol with minor modification (18, 19). We acquired a custom gRNA library from GenScript, which included a single genomic locus tiling hg19 Chr3: 22,500 kb – 24,500 kb (92,683 gRNAs with MIT Specificity score > 50) and nontargeting gRNAs (4,082 gRNAs from Weissman Nontargeting pool and 3,673 gRNAs targeting 70 randomly nonexpressed gene promoter region) in the same pool (Supplemental Data File). The sgRNA libraries were constructed into the sgOpti vector (Addgene) using the NEBuilder HiFi DNA Assembly kit (NEB, E5520). To create a dox-inducible KRAB-dCas9–expressing hMSC line, we performed lentiviral infection using the pCW-KRAB-dCas9-BSD-BFP vector and selected positive cells with blasticidin (10 μg/mL). We then transduced the gRNA libraries into the hMSC line at a low multiplicity of infection (MOI; ~0.3 MOI) and selected the positive cells with puromycin (2.5 μg/mL) for 4 days. After selection, we collected positive cells using 0.05% trypsin-EDTA and reseeded them into new culture dishes. The cells were grown to confluence with 0.2 μg/mL puromycin and 1 μg/mL doxycycline. Ultimately, we collected 320 million cells for analyses.

We utilized the PrimeFlow RNA Assay Kit (Thermo Fisher Scientific, 88-18005) to perform flow cytometry selection according to the manufacturer’s instructions. For each sample, 80 million cells were labeled with probesets targeting the mRNAs of genes of interest and the positive control, GAPDH. Probesets used are listed in Supplemental Table 2. We diluted the stained cells in PBS with 0.5% BSA to a concentration of 2 × 10^7^ cells/mL, filtered them using a 30 μm filter, and sorted 80 million cells for each screen into 3 bins based on the fluorescence intensity of the target genes using the BD FACSAria II. To control for differences in staining efficiency, we normalized the fluorescence associated with UBE2E2 or UBE2E1 to GAPDH. We set the gates for each bin on the compensated signal to capture cells according to the following percentiles: low, 0%–15%; center, 35%–65%; high, 85%–100%.

We collected each sorted cell sample by centrifugation at 800*g* for 5 minutes and extracted genomic DNA using the Qiagen DNeasy Blood & Tissue kit (Qiagen, 69516) with minor modifications to the cell lysis procedure. Briefly, we added 360 μL of Qiagen ATL buffer and 40 μL of Qiagen proteinase K to the lysis buffer and mixed thoroughly by vortexing for 15 seconds. The cell lysate was incubated on a Thermomixer at 60°C with 400 rpm vibration overnight. We then added 400 μL of a Qiagen AL buffer mixture (398 μL AL buffer plus 2 μL carrier RNA), mixed the lysis by inverting it 10 times, and added 4 μL of RNase mixture (Thermo Fisher Scientific) followed by incubation at 37°C for 30 minutes to remove RNA contamination. After adding 400 μL ethanol and mixing thoroughly by vortexing, we purified the genomic DNA using silica-membrane–based columns according to the manufacturer’s protocol. We amplified sgRNA integrations from 1 μg of genomic DNA for each sample by PCR using indexed sgRNA sequencing library primers containing Illumina adaptors with the NEB Q5 Next Ultra II kit (Supplemental Table 3). The NGS library (~200 bp) was purified by 6% TBE gel separation and the Beckman Ampure XP kit, and the pooled library was sequenced on a NovaSeq 6000 using custom Illumina sequencing and index primers (Supplemental Table 4) to an average depth of > 250 reads per sgRNA.

Bioinformatics and biostatistical analysis were performed to quantify gRNA amplicons. First, based on the raw sgRNA sequencing data, we conducted text searching to extract the 15 bp sequences upstream of the GTTTAAGAGCTATGCTGGAA sequences (the common downstream sequence of the different CRISPRi gRNAs) and quantified their abundance per sample. Second, according to the gRNA library profile (Supplemental Data File including No express, Tiling and Weissman off-target regions), we calculated the number of reads per gRNA per sample, and the gRNA abundance was further normalized by the total count. Next, based on gRNA coordinate position, gRNAs were grouped by a 2,000 bp sliding window strategy, where neighboring gRNAs locates within the window region were clustered together. Finally, differential analysis was performed to screen the windows with differential gRNA abundance when comparing bins of differential *UBE2E2* and *UBE2E1* expression (based on PrimeFlow signal). These gRNAs were further annotated by TSS and exon regions and overlapped with the ATAC-Seq peak regions.

### Proteomics.

3T3L1 cell lines with *Ube2e1* or *Ube2e2* knockdown were treated with or without an adipogenic cocktail, containing dexamethasone (1 μM), insulin (10 μg/mL), and isobutyl-methylxanthine (0.5 mM), for 24 hours. We collected cell pellets using 1% trypsin-EDTA. TMT-based quantitative proteomics analysis was performed by Creative Proteomics (Creative Proteomics). The TMT10plex Isobaric Label Reagent Set and the Pierce Quantitative Colorimetric Peptide Assay were purchased from Thermo Fisher Scientific. Labeled peptides from each group were fractionated into 6 components using HPLC. Nano liquid chromatography-tandem mass spectrometry (LC-MS/MS) analysis was performed using an Ultimate 3000 nano UHPLC system (Thermo Fisher Scientific) coupled online to a Q Exactive HF mass spectrometer (Thermo Fisher Scientific) equipped with a Nanospray Flex Ion Source (Thermo Fisher Scientific). For TMT-labeled samples, the full scan was performed between 350 and 1,650 *m/z* at a resolution of 120,000 at 200 Th. The automatic gain control target for the full scan was set to 3 × 10^6^. The MS/MS scan operated in Top 15 mode using the following settings: resolution of 30,000 at 200 Th, automatic gain control target of 1 × 10^5^, normalized collision energy at 32%, isolation window of 1.2 Th, charge state exclusion for unassigned, 1, or >6, and dynamic exclusion of 40 seconds. Raw MS files were analyzed and searched against the mouse protein database using MaxQuant (version 1.6.2.14). The parameters were set as follows: protein modifications included carbamidomethylation (C) as a fixed modification and oxidation (M) as a variable modification; the TMT-10plex was specified; enzyme specificity was set to trypsin; the maximum missed cleavages were set to 2; the precursor ion mass tolerance was set to 10 ppm; and MS/MS tolerance was 0.6 Da.

### RNA-Seq.

3T3L1 cell lines with Ube2e1 or Ube2e2 knockdown were treated with or without an adipogenic cocktail for 12 hours. We collected cell pellets using 1% trypsin-EDTA. Total RNA was extracted using the Qiagen RNeasy Kit (Qiagen, 74104) following the manufacturer’s instructions. RNA integrity and quantification were assessed using the Agilent 2100 Bioanalyzer. After quality control procedures, mRNAs were enriched using oligo(dT) beads, and rRNA was removed using the Ribo-Zero kit. Library construction and quality control were performed by Novogene (Novogene) following the Illumina protocol. Libraries were pooled and sequenced on the Illumina PE150 sequencing platform. Each sample achieved 20 million clean reads after filtration by FASTQC. RNA-Seq quantification was analyzed using kallisto (52). Differential expression analysis was performed using DESeq2 with the criteria of |log_2_(fold change)| ≥ 0.6 and *q* < 0.05 on the Dr. Tom platform (http://biosys.bgi.com) (BGI-Shenzhen). GSEA was performed using the Broad Institute’s GSEA algorithm platform (53) (http://software.broadinstitute.org/gsea/index.jsp). The RNA-Seq data were deposited in the GEO database under accession no. GSE268800.

### ATAC-Seq.

ATAC-Seq samples were prepared following the Omni-ATAC protocol (54). Library quality control and sequencing were performed by Novogene on a NovaSeq 6000 sequencer. ATAC-Seq data were deposited in the GEO database. Trimmomatic was applied to trim low-quality reads and adapter sequences; remaining reads aligned to human reference genome hg38 by Burrows-Wheeler Aligner. The aligned reads were further filtered by SAMtools (55), marked Duplicates were marked with Picard tools, and peak calling was performed by MACS to identify chromatin accessible regions (56). Finally, peaks were annotated by R package ChIPseeker to identify their closest genes (57).

### qPCR.

RNA was extracted using RNAzol RT (Molecular Research Center Inc., RN190) according to manufacturer’s protocol. cDNA was synthesized using the High-Capacity cDNA Reverse Transcription Kit (Applied Biosystems), and qPCR was performed using the PowerUp SYBR Green Master Mix (Applied Biosystems). The ΔΔCt method was used to calculate fold change.

### ELISA.

Serum insulin was measured by ELISA (MilliporeSigma) as previously described (50). Serum adiponectin and leptin were also measured by ELISA (R&D Systems) and expressed as the ratio of adiponectin concentration (μg/mL) over leptin concentration (ng/mL).

### Histology.

We assessed adipocyte size as we have previously published and incorporated modifications to promote consistency (58–60). After H&E-stained adipose tissue sections were delabeled, a Histology Slide Scanner (PANNORAMIC 1000; 3DHistech) was used to obtain whole slide images of each tissue section. Five different regions were randomly selected from these images using CaseViewer software. The selected images were then imported into the Fiji program, where the Adiposoft plug-in was used to automatically identify adipocytes, with manual adjustments made for cells that were either unrecognized or misidentified by the software. The cross-sectional areas of adipocytes in all 5 randomized regions were measured and recorded. All analysis procedures were conducted independently by 3 individuals in a single-blinded manner, and the results were averaged.

### Statistics.

Normally distributed data were analyzed by 2-tailed *t* test when comparing 2 experimental groups. To assess significance of genotype effects for single–time point variables in experiments in which both sexes were included, we performed 2-way ANOVA and reported *P* values for genotype effect as our a priori primary hypothesis was related to genotype. Prism 9 or 10 was used for statistical analyses unless otherwise stated. Data are shown as mean ± SD, with the exception of GTT/ITT curves, which show mean ± SEM.

### Study approval.

Animal experiments were approved by and in compliance with the Brigham and Women’s Hospital and the University of Pittsburgh IACUC.

### Data availability.

Data contained in this manuscript can either be accessed from the Supporting Data Values file or from the GEO database: GSE269375 and GSE268800.

## Author contributions

YZ designed research studies, conducted experiments, acquired data, analyzed data, provided reagents, and wrote the manuscript. NLD designed research studies, conducted experiments, and acquired data. TP conducted experiments and acquired data. REA conducted experiments and acquired data. GVNK conducted experiments and acquired data. HC acquired data. WQ acquired data. JY acquired data. KP acquired data. TA acquired data. AXS acquired data. SL analyzed data. MLS designed research studies, conducted experiments, acquired data, analyzed data, and wrote the manuscript.

## Supplementary Material

Supplemental data

Unedited blot and gel images

Supporting data values

## Figures and Tables

**Table 1:**
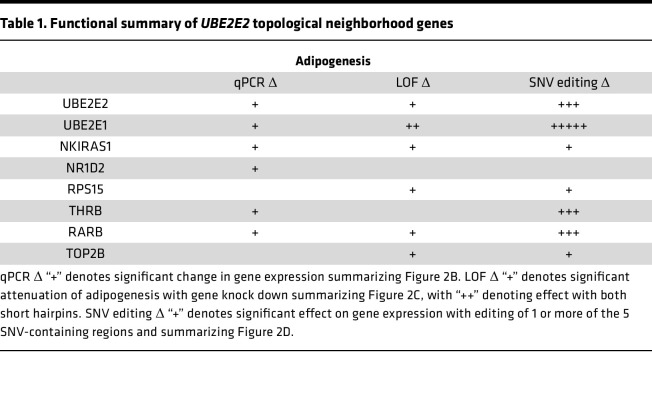
Functional summary of *UBE2E2* topological neighborhood genes

**Table 2:**
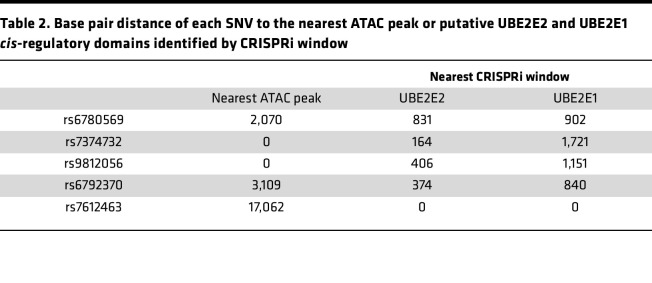
Base pair distance of each SNV to the nearest ATAC peak or putative UBE2E2 and UBE2E1 *cis*-regulatory domains identified by CRISPRi window

## Version 1 12/10/2024

In-Press Preview

## Version 2 01/23/2025

Electronic publication

## References

[1] Willemsen G (2015). The concordance and heritability of type 2 diabetes in 34,166 twin pairs from international twin registers: the discordant twin (DISCOTWIN) Consortium. Twin Res Hum Genet.

[2] Bonnefond A, Froguel P (2015). Rare and common genetic events in type 2 diabetes: what should biologists know?. Cell Metab.

[3] Vujkovic M (2020). Discovery of 318 new risk loci for type 2 diabetes and related vascular outcomes among 1.4 million participants in a multi-ancestry meta-analysis. Nat Genet.

[4] Narayan KM (2007). Effect of BMI on lifetime risk for diabetes in the U.S. Diabetes Care.

[5] Fazeli PK (2020). Aging is a powerful risk factor for type 2 diabetes mellitus independent of body mass index. Gerontology.

[6] Gómez-Ambrosi J (2011). Body adiposity and type 2 diabetes: increased risk with a high body fat percentage even having a normal BMI. Obesity (Silver Spring).

[7] Emdin CA (2017). Genetic association of waist-to-hip ratio with cardiometabolic traits, type 2 diabetes, and coronary heart disease. JAMA.

[8] Racette SB (2006). Abdominal adiposity is a stronger predictor of insulin resistance than fitness among 50-95 year olds. Diabetes Care.

[9] Fox CS (2007). Abdominal visceral and subcutaneous adipose tissue compartments: association with metabolic risk factors in the Framingham Heart Study. Circulation.

[10] Weyer C (2000). Enlarged subcutaneous abdominal adipocyte size, but not obesity itself, predicts type II diabetes independent of insulin resistance. Diabetologia.

[11] Lee JJ (2016). Association of changes in abdominal fat quantity and quality with incident cardiovascular disease risk factors. J Am Coll Cardiol.

[12] Chu AY (2017). Multiethnic genome-wide meta-analysis of ectopic fat depots identifies loci associated with adipocyte development and differentiation. Nat Genet.

[13] Yamauchi T (2010). A genome-wide association study in the Japanese population identifies susceptibility loci for type 2 diabetes at UBE2E2 and C2CD4A-C2CD4B. Nat Genet.

[14] Gupta RM (2017). A genetic variant associated with five vascular diseases is a distal regulator of endothelin-1 gene expression. Cell.

[15] Claussnitzer M (2015). FTO obesity variant circuitry and adipocyte browning in humans. N Engl J Med.

[16] Stacey D (2019). ProGeM: a framework for the prioritization of candidate causal genes at molecular quantitative trait loci. Nucleic Acids Res.

[17] Zhu Z (2016). Integration of summary data from GWAS and eQTL studies predicts complex trait gene targets. Nat Genet.

[18] Fulco CP (2016). Systematic mapping of functional enhancer-promoter connections with CRISPR interference. Science.

[19] Fulco CP (2019). Activity-by-contact model of enhancer-promoter regulation from thousands of CRISPR perturbations. Nat Genet.

[20] Liu T (2019). TADKB: Family classification and a knowledge base of topologically associating domains. BMC Genomics.

[21] Frühbeck G (2018). Adiponectin-leptin ratio: A promising index to estimate adipose tissue dysfunction. Relation with obesity-associated cardiometabolic risk. Adipocyte.

[22] López-Jaramillo P (2014). The role of leptin/adiponectin ratio in metabolic syndrome and diabetes. Horm Mol Biol Clin Investig.

[23] Stewart MD (2016). E2 enzymes: more than just middle men. Cell Res.

[24] Zheng N, Shabek N (2017). Ubiquitin ligases: structure, function, and regulation. Annu Rev Biochem.

[25] Buetow L, Huang DT (2016). Structural insights into the catalysis and regulation of E3 ubiquitin ligases. Nat Rev Mol Cell Biol.

[26] Metzger MB (2014). RING-type E3 ligases: master manipulators of E2 ubiquitin-conjugating enzymes and ubiquitination. Biochim Biophys Acta.

[27] Chutkow WA (2010). Deletion of the alpha-arrestin protein Txnip in mice promotes adiposity and adipogenesis while preserving insulin sensitivity. Diabetes.

[28] Kim JY (2007). Obesity-associated improvements in metabolic profile through expansion of adipose tissue. J Clin Invest.

[29] Kim SM (2014). Loss of white adipose hyperplastic potential is associated with enhanced susceptibility to insulin resistance. Cell Metab.

[30] Wang QA (2013). Tracking adipogenesis during white adipose tissue development, expansion and regeneration. Nat Med.

[31] Chen Q (2016). Fate decision of mesenchymal stem cells: adipocytes or osteoblasts?. Cell Death Differ.

[32] Gregoire FM (1998). Understanding adipocyte differentiation. Physiol Rev.

[33] Kahn CR (2019). Altered adipose tissue and adipocyte function in the pathogenesis of metabolic syndrome. J Clin Invest.

[34] Ruiz-Ojeda FJ (2016). Cell models and their application for studying adipogenic differentiation in relation to obesity: a review. Int J Mol Sci.

[35] Tang QQ, Lane MD (2012). Adipogenesis: from stem cell to adipocyte. Annu Rev Biochem.

[36] Jiang MS, Lane MD (2000). Sequential repression and activation of the CCAAT enhancer-binding protein-alpha (C/EBPalpha) gene during adipogenesis. Proc Natl Acad Sci U S A.

[37] Fathzadeh M (2020). FAM13A affects body fat distribution and adipocyte function. Nat Commun.

[38] Majithia AR (2014). Rare variants in PPARG with decreased activity in adipocyte differentiation are associated with increased risk of type 2 diabetes. Proc Natl Acad Sci U S A.

[39] Khurana E (2013). Integrative annotation of variants from 1092 humans: application to cancer genomics. Science.

[40] Lupiáñez DG (2015). Disruptions of topological chromatin domains cause pathogenic rewiring of gene-enhancer interactions. Cell.

[41] Weinhold N (2014). Genome-wide analysis of noncoding regulatory mutations in cancer. Nat Genet.

[42] Cochran JN (2020). Non-coding and loss-of-function coding variants in TET2 are associated with multiple neurodegenerative diseases. Am J Hum Genet.

[43] Turnbull C (2010). Variants near DMRT1, TERT and ATF7IP are associated with testicular germ cell cancer. Nat Genet.

[44] Weedon MN (2014). Recessive mutations in a distal PTF1A enhancer cause isolated pancreatic agenesis. Nat Genet.

[45] Chandra V (2021). Promoter-interacting expression quantitative trait loci are enriched for functional genetic variants. Nat Genet.

[46] Jazdzewski K (2008). Common SNP in pre-miR-146a decreases mature miR expression and predisposes to papillary thyroid carcinoma. Proc Natl Acad Sci U S A.

[47] Smemo S (2014). Obesity-associated variants within FTO form long-range functional connections with IRX3. Nature.

[48] Opazo JC (2015). Gene turnover in the avian globin gene families and evolutionary changes in hemoglobin isoform expression. Mol Biol Evol.

[49] Pearson JC (2005). Modulating Hox gene functions during animal body patterning. Nat Rev Genet.

[50] Zhang Y (2018). Targeting nuclear receptor NR4A1-dependent adipocyte progenitor quiescence promotes metabolic adaptation to obesity. J Clin Invest.

[51] Wang Y (2018). The 3D Genome Browser: a web-based browser for visualizing 3D genome organization and long-range chromatin interactions. Genome Biol.

[52] Bray NL (2016). Near-optimal probabilistic RNA-Seq quantification. Nat Biotechnol.

[53] Subramanian A (2005). Gene set enrichment analysis: a knowledge-based approach for interpreting genome-wide expression profiles. Proc Natl Acad Sci U S A.

[54] Corces MR (2017). An improved ATAC-Seq protocol reduces background and enables interrogation of frozen tissues. Nat Methods.

[55] Li H, Durbin R (2009). Fast and accurate short read alignment with Burrows-Wheeler transform. Bioinformatics.

[56] Danecek P (2021). Twelve years of SAMtools and BCFtools. Gigascience.

[57] Yu G (2015). ChIPseeker: an R/Bioconductor package for ChIP peak annotation, comparison and visualization. Bioinformatics.

[58] Monji A (2022). A cycle of inflammatory adipocyte death and regeneration in murine adipose tissue. Diabetes.

[59] Chen HC, Farese RV (2002). Determination of adipocyte size by computer image analysis. J Lipid Res.

[60] Parlee SD (2014). Quantifying size and number of adipocytes in adipose tissue. Methods Enzymol.

