# Supplementary material for: Noncoding variation near UBE2E2 orchestrates cardiometabolic pathophenotypes through polygenic effectors
Source: JCI Insight. 2024 Dec 10;10(2):e184140. doi: 10.1172/jci.insight.184140 (PMC11790016; doi:10.1172/jci.insight.184140)
Supplement: Supplemental data [file jciinsight-10-184140-s179.pdf]

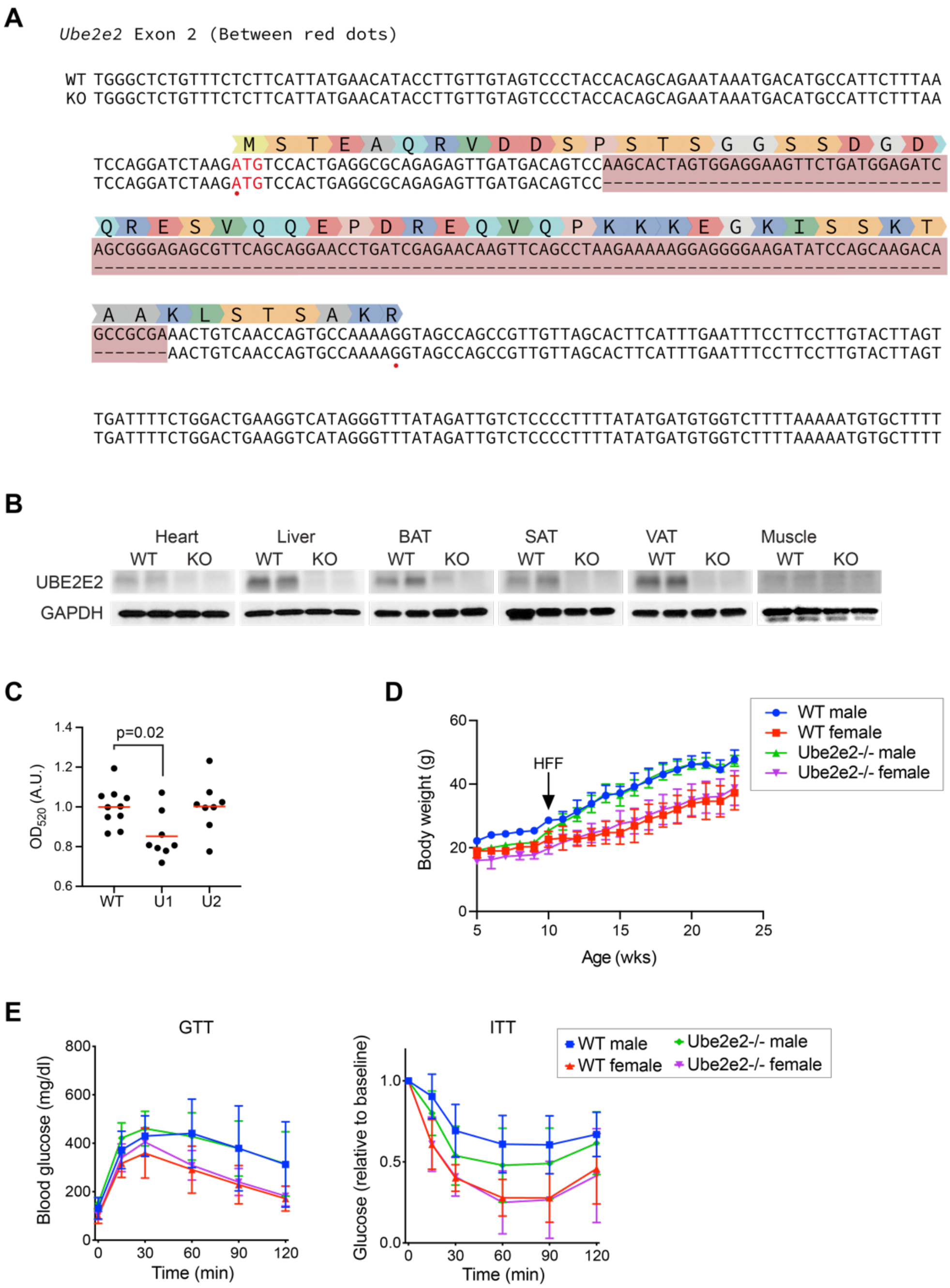

Figure S1. Ube2e2 knockout mouse model.
(A) Sequencing result demonstrating successful CRISPR targeting of exon 2 of *Ube2e2*.
(B) Western blot analyses of multiple tissue from *Ube2e2-/-* mice (KO) versus wildtype (WT) mice. Antibodies used were: UBE2E2 (Invitrogen, PA5-42363) and GAPDH (6C5 Santa Cruz, sc-32233).
(C) *Ex vivo* adipogenesis assay of primary subcutaneous adipocyte progenitors isolated from wild-type mice, the putative knockout line (U1) and a second CRISPR line (U2) that had a simple point mutation. Each dot indicates assessment of adipogenesis in progenitors isolated from a single mouse. Significance assessed by one way ANOVA, Dunnett's test, n=8-10 mice.
(D) Body weight curves for wild-type (WT) and the U1 knockout line (Ube2e2$^{-/-}$) with high fat feeding (HFF) initiated at 10 weeks of age, n=8-9. Data shown as mean +/- s.d.m.
(E) Glucose tolerance testing (GTT) and insulin tolerance testing (ITT) of *Ube2e2*$^{-/-}$ mice and wildtype mice after 12 weeks of high fat feeding, starting at 10 weeks of age (n=13-14). Area under the curve (a.u.c.) data shown in Figure 1. No significant differences were detected (mean +/- s.d.m.).

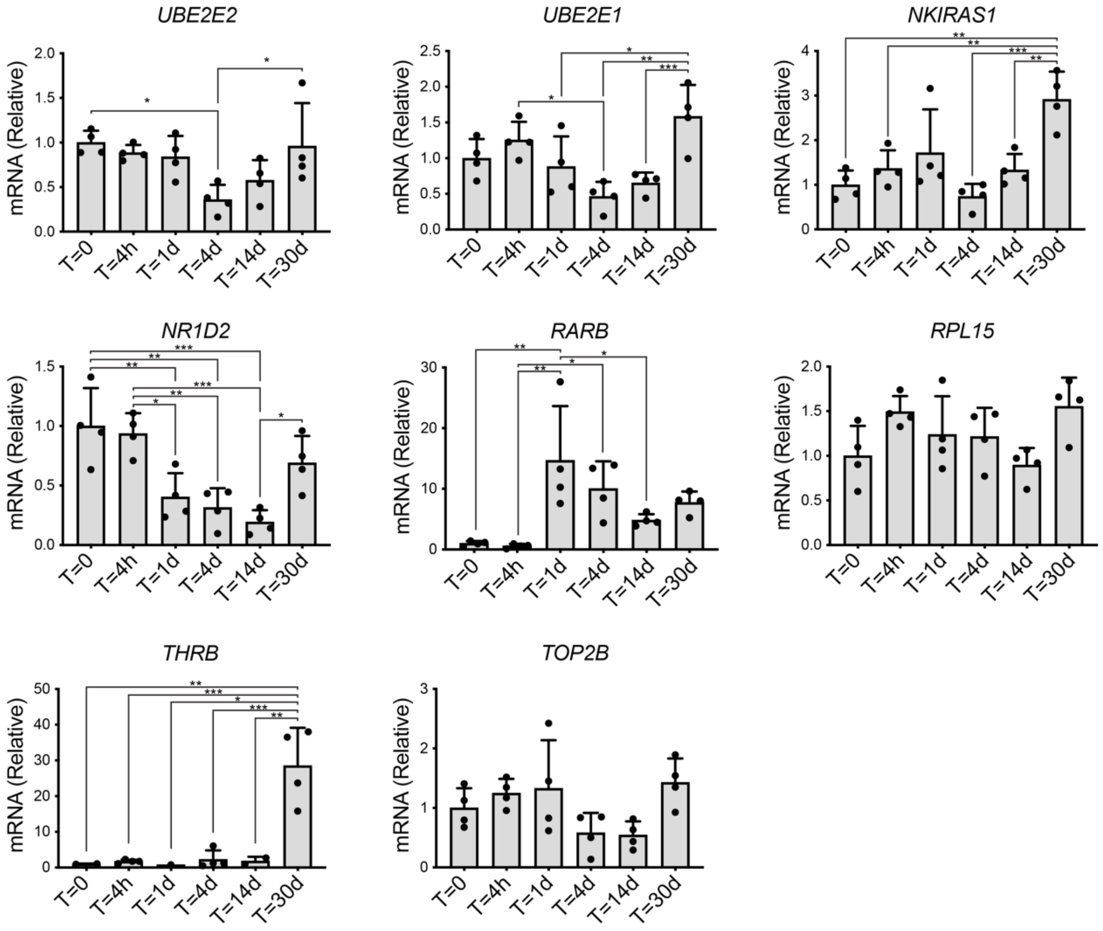

Figure S2. Dynamic expression of genes in the topological neighborhood of *UBE2E2* during adipogenesis of mesenchymal stem cells (MSC).
MSC were subjected to adipogenic differentiation for 30 days with harvesting of cell homogenenates for qPCR at baseline, 4h, 1d, 4d, 14d, and 30d after adipogenic induction. Significance assessed by one-way ANOVA with Tukey's multiple comparison test: *p<0.05, **p<0.01, ***p<0.005. These data formed the basis for Figure 2B heatmap.

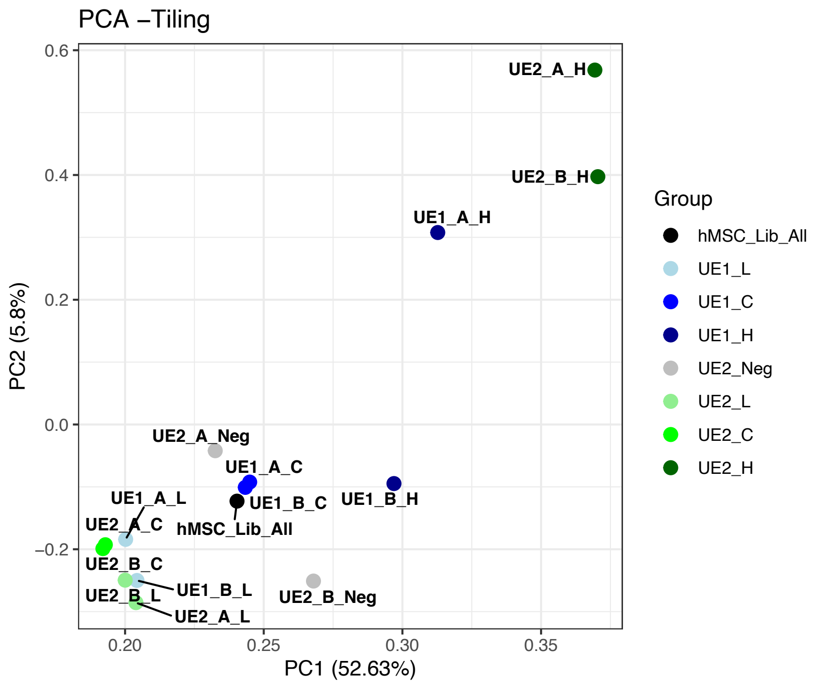

Figure S3. gRNA sequencing discriminates MSC isolated by degree of UBE2E2/E1 expression. gRNA library tiling across the UBE2E2 and UBE2E1 loci was introduced to MSC in culture. In situ mRNA probes were used to sort by high, low, and medium levels of *UBE2E2* and *UBE2E1* expression and then cells were sequenced to identify regions of gRNA enrichment/depletion. A sliding window approach was used to identify putative regulatory hotspots. PCA plot of these data demonstrate discrimination of high/low/medium expression MSC.

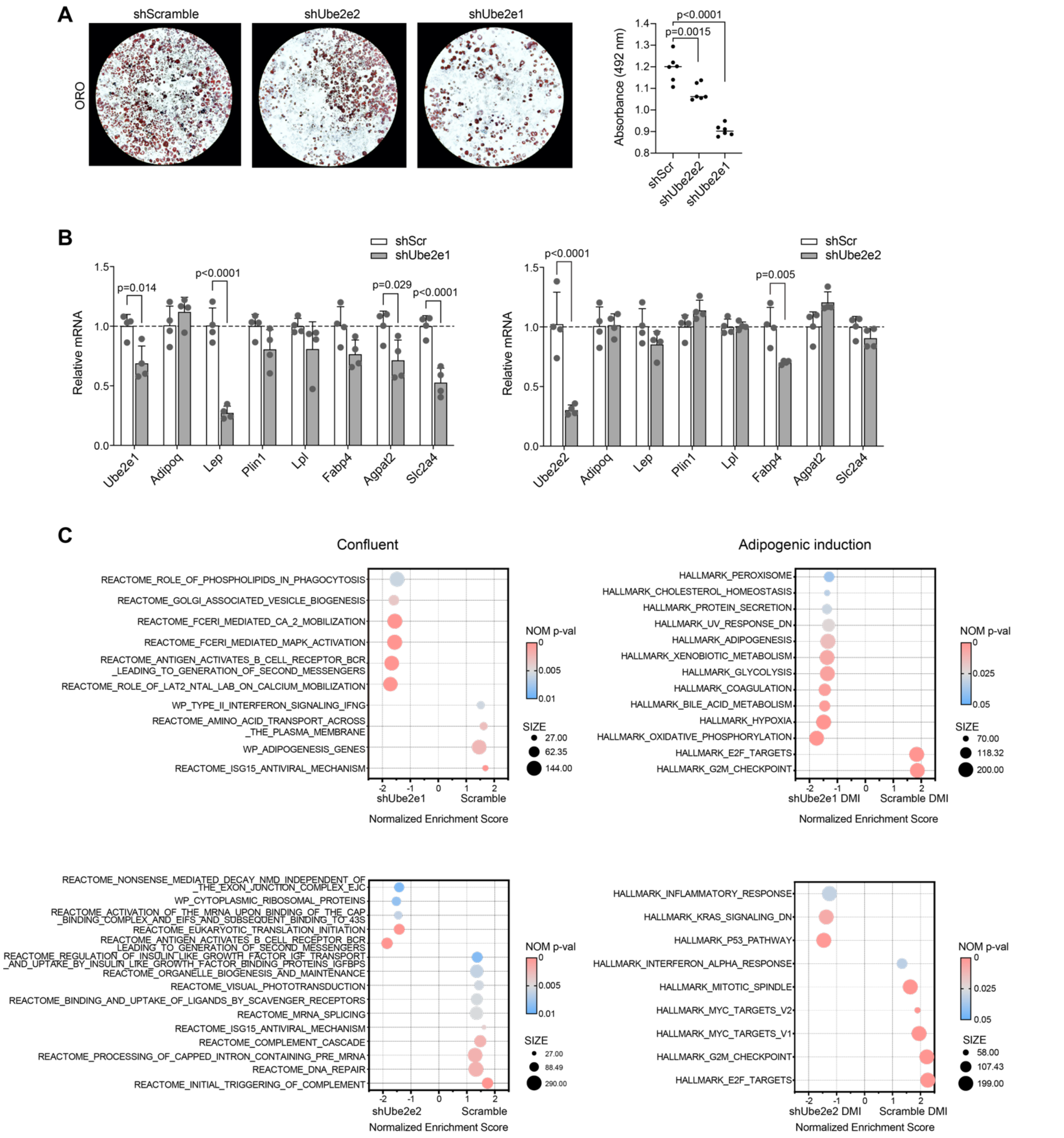

Figure S4. Gene set enrichment analyses for Ube2e2 and Ube2e1 loss of function in 3T3L1 cells.
(A) 3T3L1 adipogenesis assay (oil-red-o) with Ube2e1 and Ube2e2 loss of function via sh-RNA and ORO staining. One way ANOVA, Dunnett's multiple comparison test, n=6 technical replicates.
(B) qPCR assessment of adipogenic genes after adipogenic differentiation as in A. Two-way ANOVA with Sidak's multiple comparison test, n=4 technical replicates.
(C) RNA-seq was performed on confluent 3T3L1 cells (left) and 12 hours after induction of adipogenic differentiation (right). Ube2e1 knockdown (top) or Ube2e2 knockdown (bottom) are compared to control cells transduced with sh-scramble. GSEA analyses are shown as bubble plots with nominal p-value indicated by the color scale, size indicated by bubble size, and plotted as a function of the normalized enrichment score (x-axis). These analyses complement the transcriptomics data shown in Figure 4.

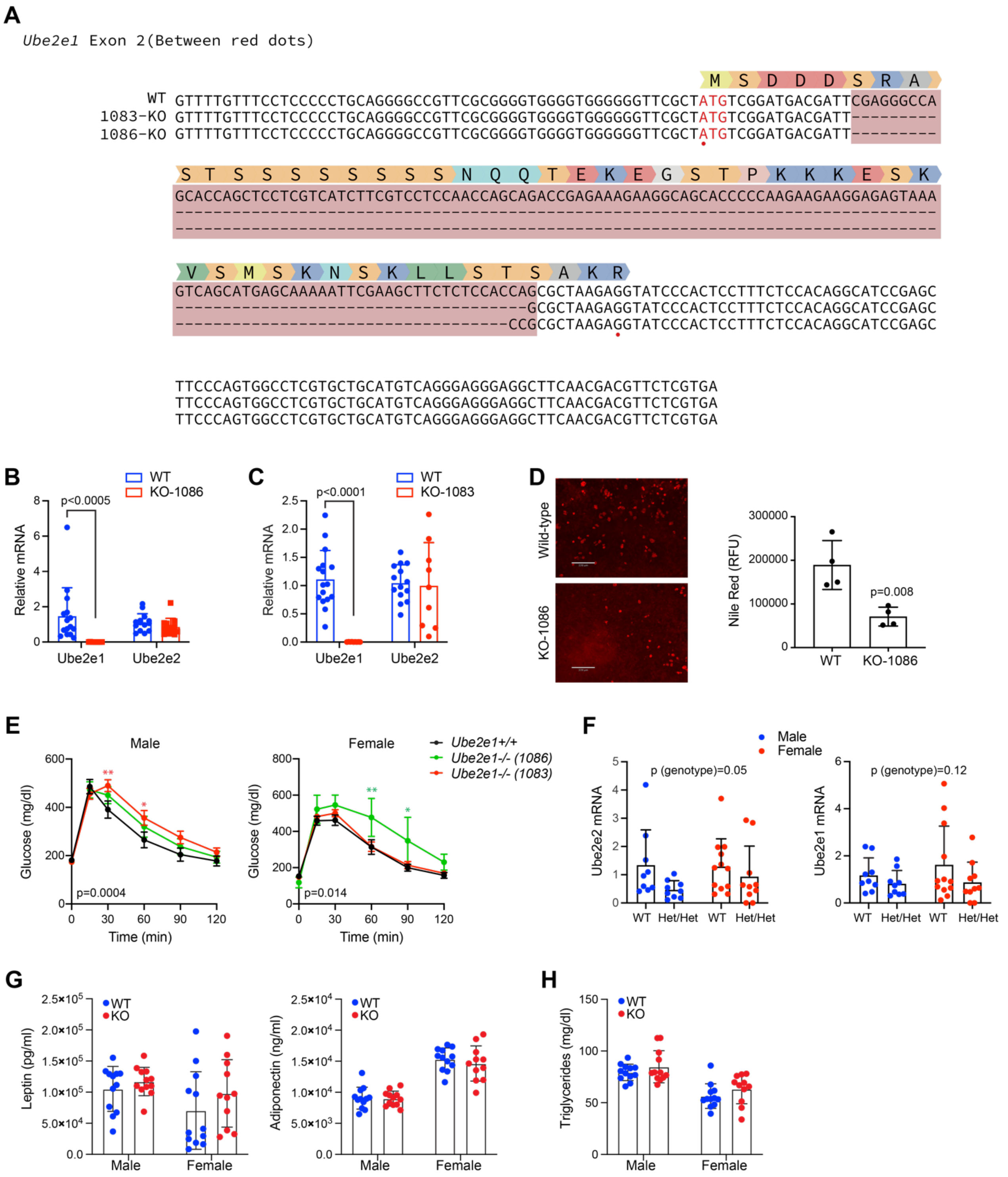

Figure S5. Ube2e1 knockout mouse model.

(A) Sequencing result demonstrating successful CRISPR targeting of exon 2 of *Ube2e1*.

(B) qPCR analysis of adipose tissues from the 1086 knockout line relative to wildtype and demonstrating loss of *Ube2e1* mRNA but not *Ube2e2* mRNA. Significance assessed by ANOVA and Sidak's correction, n=11-15.

(C) qPCR analysis of adipose tissues from the 1083 knockout line relative to wildtype and demonstrating loss of *Ube2e1* mRNA but not *Ube2e2* mRNA. Significance assessed by ANOVA and Sidak's correction, n=9-16.
(D) *Ex vivo* adipogenesis assay of primary subcutaneous adipocyte progenitors isolated from wild-type mice versus the 1086 Ube2e1 knockout line (n=4 mice per group). Significance assessed by two-sided t-test. Representative Nile red images shown, scale bar = 320μm.
(E) Glucose tolerance testing (GTT) of *Ube2e1*$^{-/-}$ mice and wildtype mice after 12 weeks of high fat feeding. Mean +/- s.d.m. shown. Significance assessed by two-way ANOVA, Dunnett's multiple comparison test. Note: in these early cohorts the sample size was imbalanced particularly for the 1086 line: 1086 female n=2; 1086 male n=6; 1083 female n=13; 1083 male n=15; wildtype female n=6; wildtype male n=11. Two-way ANOVA p-value is displayed with color coded symbol for Dunnett's multiple comparison test *p<0.05; **p<0.01.
(F) Subcutaneous adipose tissues from compound heterozygous Ube2e1$^{+/-}$; Ube2e2$^{+/-}$ mice were compared to wildtype control by qPCR and showing approximate halving of mRNA for both transcripts, n=9-12.
(G) Leptin and adiponectin raw values used to calculate Adiponectin:Leptin ratio shown in Figure 6H.
(H) Serum triglycerides from Figure 6 diet-induced obesity study, (n=11-12).

Supplemental Table 1. Comparison of sh-RNA knockdown efficiency to adipogenesis effect in 3T3L1 cells from Figure 2C. mRNA that were not consistently detected after shRNA knockdown are denoted as ‘u.d.’. Mean adipogenesis effect is from Figure 2C.

Hairpin	Knockdown efficiency	Adipogenesis effect (mean)
Ube2e2	0.039 ± 0.005	0.47
Thrb-a	u.d.	1.13
Thrb-b	u.d.	1.1
Rpl15-a	0.643 ± 0.063	0.36
Rpl15-b	0.387 ± 0.079	0.53
Nrld2-a	0.236 ± 0.039	0.57
Nrld2-b	0.070 ± 0.018	0.99
Rarb-a	u.d.	0.50
Rarb-b	u.d.	1.0
Top2b-a	0.260 ± 0.052	0.47
Top2b-b	0.137 ± 0.020	0.55
Nkiras-a	0.206 ± 0.037	1.06
Nkiras-b	0.453 ± 0.122	0.57
Ube2e1-a	0.117 ± 0.024	0.73
Ube2e1-b	0.273 ± 0.044	0.69

Supplemental Table 2. sgRNAs for Ube2e2 and Ube2e2 knockout mice generation.

Gene	gRNA 1	gRNA 2
Ube2e2	AGCACTAGTGGAGGAAGTTC	CGAAACTGTCAACCAGTGCC
Ube2e1	TTTCACAGTGCTGGTCCCAA	CCAAAGGTAAGGAGTCTCCA

Supplemental Table 3. RNA probesets for Prime Flow experiments.

Targeting RNA	Fluorochrome label	Excitation (nm)	filter (nm)	Probe set type
UBE2E2	Alexa Fluor 568	561	610/20	Type 10
UBE2E1	Alexa Fluor 488	488	530/30	Type 4
GAPDH	Alexa Fluor 750	633, 635	780/60	Type 6

Supplemental Table 4. Primers used in CRISPRi screening.

Name	Oligo Sequence
Custom sgRNA Library Oligonucleotide	TCCAATGTCCCACGACGTATCTTGTGGAAAGGACGAAACACCGNNNNNNNNNNNNNNNNNNNN[1] GTTTAAGAGCTATGCTGGAAACAGCATAGGCCAAAACCCTCCGATG
sgRNA Library Fwd	GGCTTTATATATCTTGTGGAAAGGACGAAACACCG
sgRNA Library Rev	CTTATTTAAACTTGCTATGCTGTTTCCAGCATAGCTCTTAAAC
sgRNA Sequencing Library Fwd	AATGATACGGCGACCACCGAGATCTACACNNNNCGATTTCTTGGCTTTATATATCTTGTG[2]
sgRNA Sequencing Library Rev	CAAGCAGAAGACGGCATACGAGATNNNNNNNNCGGTGCCACTTTTTCAAGTTG[3]
Illumina Sequencing Primer	CGATTTCTTGGCTTTATATATCTTGTGGAAAGGACGAAACACCG
Illumina Index Sequencing Primer	AAGGCTAGTCCGTTATCAACTTGAAAAAGTGGCACCG

[1]NNNNNNNNNNNNNNNNNNNN denotes sgRNA sequence
[2]NNNN denotes sequencing index
[3]NNNNNNNN denotes sequencing index as shown in Supplemental Table 4.

Supplemental Table 5. Primers for gRNA amplicons' NGS library preparation.

Sample	NGS Library Primer	Sequence	i7 Index sequence
All	Universal Fwd	AATGATACGGCGACCACCGAGATCTACACCGATTTCTTGGCTTTATATATCTTGTG	
UE1_Rep1_L	Rev_1	CAAGCAGAAGACGGCATACGAGATCTAGTACGCGGTGCCACTTTTTCAAGTTG	CGTACTAG
UE1_Rep1_M1	Rev_2	CAAGCAGAAGACGGCATACGAGATTTCTGCCTCGGTGCCACTTTTTCAAGTTG	AGGCAGAA
UE1_Rep1_M2	Rev_3	CAAGCAGAAGACGGCATACGAGATGCTCAGGACGGTGCCACTTTTTCAAGTTG	TCCTGAGC
UE1_Rep1_H	Rev_4	CAAGCAGAAGACGGCATACGAGATAGGAGTCCCGGTGCCACTTTTTCAAGTTG	GGACTCCT
UE1_Rep2_L	Rev_5	CAAGCAGAAGACGGCATACGAGATCATGCCTACGGTGCCACTTTTTCAAGTTG	TAGGCATG
UE1_Rep2_M	Rev_6	CAAGCAGAAGACGGCATACGAGATGTAGAGAGCGGTGCCACTTTTTCAAGTTG	CTCTCTAC
UE1_Rep2_H	Rev_7	CAAGCAGAAGACGGCATACGAGATCCTCTCTGCGGTGCCACTTTTTCAAGTTG	CAGAGAGG
UE2_Rep1_L	Rev_8	CAAGCAGAAGACGGCATACGAGATAGCGTAGCCGGTGCCACTTTTTCAAGTTG	GCTACGCT
UE2_Rep1_M	Rev_9	CAAGCAGAAGACGGCATACGAGATCAGCCTCGCGGTGCCACTTTTTCAAGTTG	CGAGGCTG
UE2_Rep1_H	Rev_10	CAAGCAGAAGACGGCATACGAGATTGCCTCTTCGGTGCCACTTTTTCAAGTTG	AAGAGGCA
UE2_Rep2_L	Rev_11	CAAGCAGAAGACGGCATACGAGATTCATGAGCCGGTGCCACTTTTTCAAGTTG	GCTCATGA
UE2_Rep2_M	Rev_12	CAAGCAGAAGACGGCATACGAGATCCTGAGATCGGTGCCACTTTTTCAAGTTG	ATCTCAGG
UE2_Rep2_H	Rev_13	CAAGCAGAAGACGGCATACGAGATTAGCGAGTCGGTGCCACTTTTTCAAGTTG	ACTCGCTA
