# Supplementary material for: Noncoding variation near UBE2E2 orchestrates cardiometabolic pathophenotypes through polygenic effectors
Source: JCI Insight. 2024 Dec 10;10(2):e184140. doi: 10.1172/jci.insight.184140 (PMC11790016; doi:10.1172/jci.insight.184140)

18-37, 38 Ube2e2 WT
18-43, 44 Ube2e2 KO

UBE2E2 anti-Rabbit, polyclonal antibody, ~25kd
GAPDH anti-mouse, monoclonal antibody, 37kd

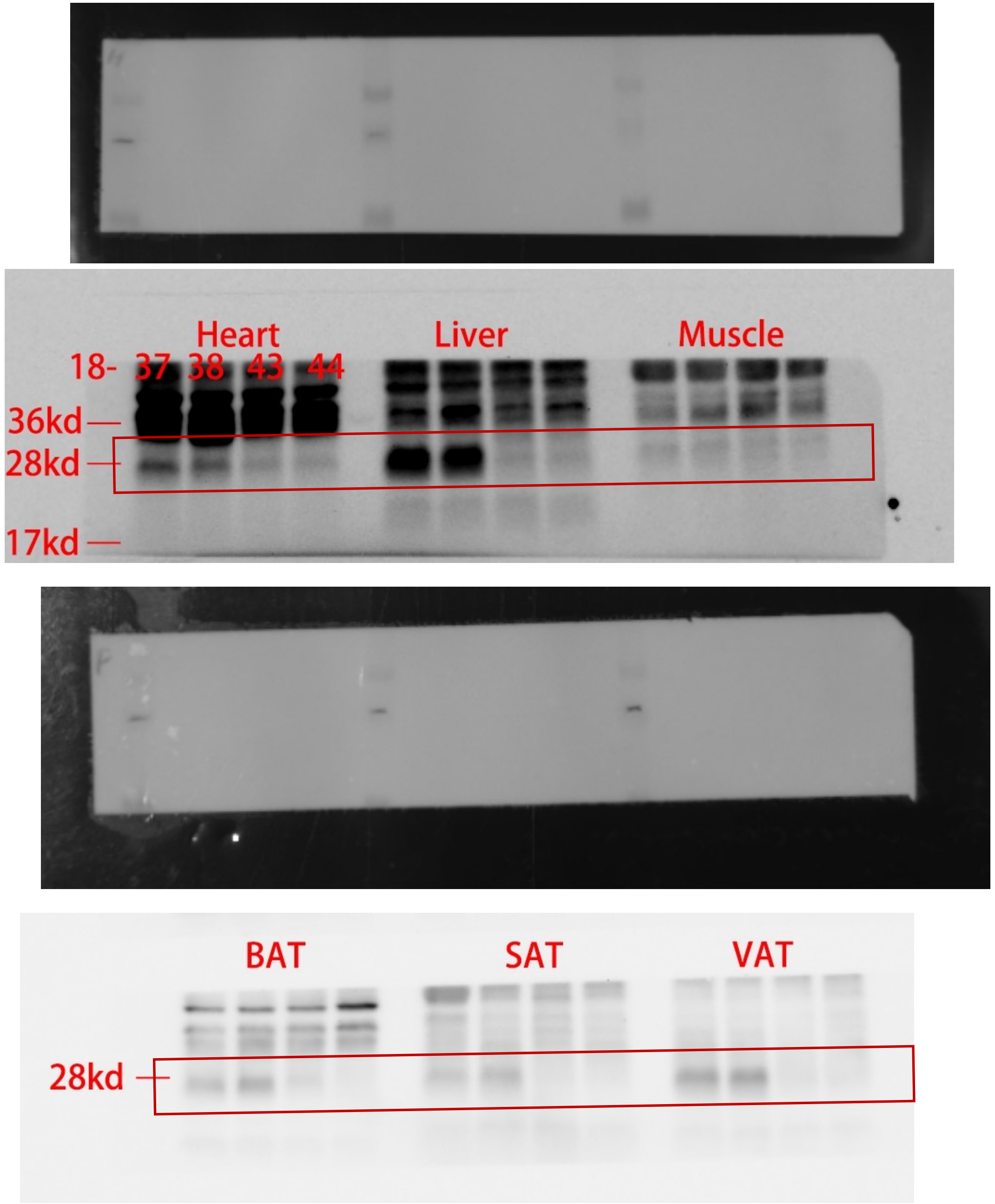

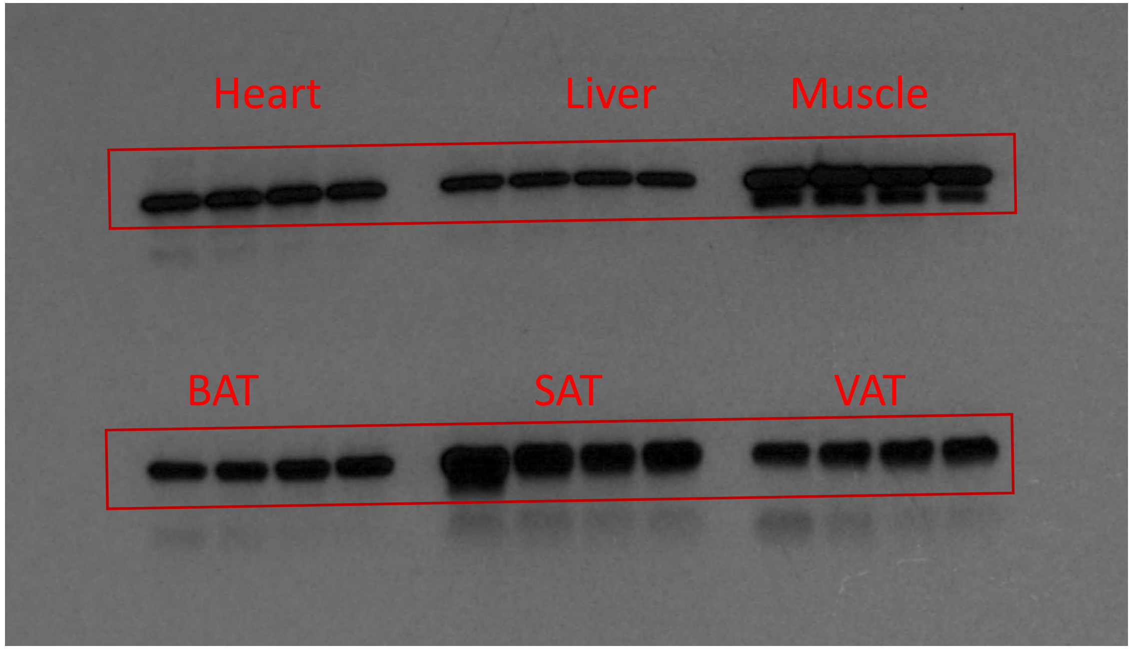

Supplement: Unedited blot and gel images [file jciinsight-10-184140-s180.pdf]
